# Genetic characteristics of 234 Italian patients with macular and cone/cone-rod dystrophy

**DOI:** 10.1038/s41598-022-07618-1

**Published:** 2022-03-08

**Authors:** Benedetto Falsini, Giorgio Placidi, Elisa De Siena, Pietro Chiurazzi, Angelo Maria Minnella, Maria Cristina Savastano, Lucia Ziccardi, Vincenzo Parisi, Giancarlo Iarossi, Marcella Percio, Barbora Piteková, Giuseppe Marceddu, Paolo Enrico Maltese, Matteo Bertelli

**Affiliations:** 1 Ophthalmology Unit, Fondazione Policlinico Universitario “A. Gemelli” IRCCS/Universita’ Cattolica del S. Cuore, Rome, Italy; 2 UOC Genetica Medica, Fondazione Policlinico Universitario “A. Gemelli” IRCCS & Istituto di Medicina Genomica, Universita’ Cattolica del S. Cuore, Rome, Italy; 3 IRCCS Fondazione Bietti, Rome, Italy; 4 Ophthalmology Unit, Bambino Gesu’ Children’s Hospital, IRCSS, Rome, Italy; 5 Department of Pediatrics, National Institute of Children’s Diseases, Faculty of Medicine, Comenius University, Bratislava, Slovak Republic; 6 MAGI’S Lab s.r.l., Via Delle Maioliche 57/D, 38068 Rovereto, TN Italy; 7 MAGI Euregio s.c.s., Bolzano, Italy

**Keywords:** Genetics, Diseases, Molecular medicine

## Abstract

Two-hundred and thirty-four Italian patients with a clinical diagnosis of macular, cone and cone-rod dystrophies (MD, CD, and CRD) were examined using next-generation sequencing (NGS) and gene sequencing panels targeting a specific set of genes, Sanger sequencing and—when necessary—multiplex ligation-dependent probe amplification (MLPA) to diagnose the molecular cause of the aforementioned diseases. When possible, segregation analysis was performed in order to confirm unsolved cases. Each patient’s retinal phenotypic characteristics were determined using focal and full-field ERGs, perimetry, spectral domain optical coherence tomography and fundus autofluorescence. We identified 236 potentially causative variants in 136 patients representing the 58.1% of the total cohort, 43 of which were unpublished. After stratifying the patients according to their clinical suspicion, the diagnostic yield was 62.5% and 53.8% for patients with MD and for those with CD/CRD, respectively. The mode of inheritance of all cases confirmed by genetic analysis was 70% autosomal recessive, 26% dominant, and 4% X-linked. The main cause (59%) of both MD and CD/CRD cases was the presence of variants in the *ABCA4* gene, followed by variants in *PRPH2* (9%) and *BEST1* (6%). A careful morpho-functional evaluation of the phenotype, together with genetic counselling, resulted in an acceptable diagnostic yield in a large cohort of Italian patients. Our study emphasizes the role of targeted NGS to diagnose MDs, CDs, and CRDs, as well as the clinical usefulness of segregation analysis for patients with unsolved diagnosis.

## Introduction

Macular, Cone and Cone-Rod Dystrophies (from now on MDs, CDs, and CRDs) are a group of Inherited Retinal Dystrophies (IRDs) caused by genetic variants in about 50 different genes.

Although these diseases have in common the involvement of primary cone photoreceptors with a predominant macular damage, they display a wide phenotypic and genetic heterogeneity, as discovered in the last two decades using modern molecular biology techniques, such as Next-Generation Sequencing (NGS) and MLPA analysis.

Targeted NGS and—when required—Whole Exome Sequencing^1^ allowed experts in this field to diagnose a high number of underestimated IRDs and, at the same time, to learn about new disease-causing genes^2,3^.

Sometimes, variants in the same gene may result in different clinical entities presenting specific differences, which can be detected by using psychophysical tests and a morpho-functional evaluation. Understanding how genetic variants in the same gene can cause a maculopathy rather than a cone-rod dystrophy (or retinitis pigmentosa), or vice versa, is still a debated issue among researchers^4,5^. Similarly, it is not completely clear why variants in the same portion of a gene may cause autosomal retinal dystrophies that can be both dominant and recessive^6–8^.

Phenotypic variability, which often recurs at interfamilial level^9^ as well as within the same families^10–13^, is also being debated in literature. It should also be appreciated that the phenotype of these conditions evolves with the age of the patient and may unpredictably progress from an early onset macular degeneration to a later and more severe cone or cone-rod dystrophy^14^. For these reasons, at present many ophthalmologists cannot easily determine a prognosis for all their patients. Moreover, given the large number of genes involved in MD and CD/CRD with overlapping clinical findings, it is difficult to distinguish a certain kind of IRDs from another.

In order to estimate the prevalence of mutated genes causing MD, CD, or CRD and to identify novel variants, with additional information regarding inheritance patterns and genotype–phenotype correlations, we studied a cohort of 234 Italian patients, analyzing their genetic and clinical characteristics.

## Materials and methods

This clinical study was carried out in compliance with the ICH Guidelines for Good Clinical Practice and following the requirements of the Declaration of Helsinki (1991). Written informed consents for clinical and molecular testing were obtained from all the adult subjects or from the underage patients’ parents. The study was approved by the Ethical Committee of Azienda Sanitaria dell’Alto Adige, Italy (Approval No. 132-2020).

### Patients

In this retrospective study, we collected data from 234 Italian patients (age range 7–88 years; female/male ratio = 0.92) who had received a clinical diagnosis of MD, CD, or CRD between the years 2009 and 2019. Moreover, family segregation studies were carried out on a total of 80 relatives, 62 healthy and 18 affected, from 41 different families. Patients were followed at the Ophthalmology and Medical Genetics Units of the Agostino Gemelli Hospital (Università Cattolica del Sacro Cuore, Rome, Italy), Fondazione Bietti per l’Oftalmologia IRCCS (Rome, Italy), and at the Ophthalmology Unit of Bambino Gesù Children’s Hospital (Rome, Italy).

The diagnosis was based on family history, age of onset, clinical findings, retinal imaging, and ERG abnormalities.

Each subject in this study was clinically evaluated, including the probands’ family members, both those affected and those unaffected. Probands’ relatives who presented no evidence of the disease underwent a full ophthalmic evaluation including dilated fundus examination. Affected subjects underwent a complete ophthalmological examination, including best-corrected visual acuity (BCVA), anterior segment biomicroscopy, intraocular pressure measurement, direct and indirect ophthalmoscopy with 15D noncontact lens (Volk); standard 30-2 static and semi-automated kinetic perimetry (Octopus 900, Haag Streit, USA); color fundus photos and fundus autofluorescence (FAF; Daytona wide-field retinography); spectral-domain optical coherence tomography (SD-OCT) (Heidelberg Spectralis OCT2, Heidelberg Engineering, Heidelberg, Germany); full-field ERG recording according to the International Society for Clinical Electrophysiology of Vision (ISCEV) standards (retimax recording system, CSO, Florence, Italy) and Focal ERG from the central 18° region.

BCVA values were obtained using Early Treatment Diabetic Retinopathy Study (ETDRS) charts, in compliance with common clinical standards.

The main outcome measure to differentiate among MD, CD, and CRD was electroretinography. We classified almost all patients using a functional classification, according to the degree of impairment in full-field and focal electroretinographic (ERG) recordings^15^.

Full-field ERG (ffERG) and Focal ERG (fERG) were performed as previously described^16,17^.

In particular, for ffERG we considered the amplitude of the cone and rod-mediated B-waves. For fERG we analyzed the amplitude of the response first harmonic at 41 Hz, quantified by off-line discrete Fourier analysis. While the first diagnostic visit was carried out by following ISCEV standards for ffERG, all the subsequent visits were carried out by adhering to a modified protocol according to which the electrodes were placed on the subjects’ eyelids, which were much better tolerated by patients, particularly young ones, than corneal electrodes.

The choice of patients for this study met the following inclusion criteria: typical CRD with a cone–rod pattern of retinal dysfunction, as determined by the ISCEV standard Ganzfeld electroretinography, standard perimetry, and classic fundus appearance (as shown by retinal imaging exams); inheritance pattern unequivocally determined by a detailed family and medical history; absent or minimal ocular media opacities; absence of nystagmus; foveal fixation or preferred retinal locus for fixation within 38 of the fovea and stable throughout the follow-up; no concomitant ocular, visual, or systemic diseases.

Reduced or undetectable fERG amplitudes with a preserved full-field photopic and scotopic ERG response suggest a Macular Dystrophy.

Reduced or undetectable full-field photopic ERG response with preserved scotopic ERG indicate a Cone Dystrophy.

When both full-field photopic and scotopic ERG amplitudes are reduced or undetectable, with cone responses more severely and precociously affected than rod responses, the diagnosis of Cone-Rod Dystrophy is most likely^18,19^.

When electroretinography was questionable or unavailable, we considered retinal imaging as a benchmark.

Patients with syndromic pictures and with a suspicion of autoimmune retinopathies (AIRs) were excluded from this study. Indeed, syndromic retinopathies have typical pictures that allow a precise clinical diagnosis that may not need genetic confirmation or the use of massive sequencing and genetic panels. The differential AIRs diagnosis was made on the basis of the consensus and criteria set out in the review by Sen et al.^20^. Briefly, the diagnosis was made by exclusion in the presence of antiretinal antibodies and of a combination of clinical features including common symptoms and visual field and ERG alterations associated, in most cases, with a normal-appearing fundus. None of our patients presented such clinical manifestations or was affected by a concomitant neoplastic disease. The analysis of both of these patient groups could skew the diagnostic yield result of a genetic test for inherited diseases such as MDs, CDs and CRDs. Patients with other Inherited Retinal Dystrophies, such as retinitis pigmentosa based on rod-cone pattern, were also excluded.

### Genetic testing

Genetic testing was performed at MAGI’s laboratories (MAGI’S Lab, Rovereto, Italy, and MAGI Euregio, Bolzano-Bozen, Italy).

DNA was extracted from whole blood or saliva using a commercial kit (Blood DNA Kit E.Z.N.A.; Omega Bio-Tek Inc., Norcross, GA, USA). Sanger Sequencing was performed in 27 patients, tested between 2009 and 2013, using a Beckman Coulter CEQ 8000 sequencer (Beckmann Coulter, Milan, Italy). Among them, 19 patients were tested to detect variants in the *ABCA4* gene, depending on the clinical suspicion of Stargardt Disease or Cone Dystrophy; the other six were tested for *BEST1* (3), *ELOVL4* (1). *CRX* (1). and *GUCY2D* (1) aiming at diagnosing Best vitelliform macular dystrophy, Stargardt-like macular dystrophy, and two Cone-rod Dystrophies, respectively.

The remaining 207 patients were tested between 2014 and 2019 via targeted NGS, using gene panels that have been continuously updated over the years (Table S1).

Forty-one patients underwent second level testing when the first one was inconclusive (e.g. patients with monoallelic pathogenic variants in a recessive gene) or uninformative (with no variant explaining the phenotype). The custom NGS panel, referred to as the “Non-syndromic Inherited Retinal Dystrophies panel” and designed at MAGI’s laboratories, included 138 genes involved in the pathogenesis of retinopathies (Table S1).

NGS was performed on a MiSeq personal sequencer (Illumina, San Diego, CA), following the molecular and bioinformatic strategy that we previously published^21,22^.

Deep intronic variants were not evaluated.

*ABCA4* Multiplex ligation-dependent probe amplification (MLPA) was also performed, in order to detect possible deletions or duplications in the 16 patients that were found to carry only a single *ABCA4* variant after NGS or Sanger sequencing.

The pathogenicity of new variants was evaluated according to the American College of Medical Genetics and Genomics (ACMG) guidelines^23^, using the online tool VarSome (https://varsome.com/—accessed on November 2021)^24^. In the present work, we only reported variants that were pathogenic, likely pathogenic, or VUS (variants of unknown significance).

Before carrying out the genetic test, all patients underwent a genetic counselling, in order to reconstruct their family history and pedigree. Patients not reporting parental consanguinity and not having any evidence of other affected family members were defined as "sporadic".

Genetic testing was considered conclusive when patients had: a pathogenic and/or likely pathogenic variants in dominant and X-linked genes, even in the absence of family history; VUS variants on dominant and X-linked genes, with the support of family history or of the family segregation study; at least two variants in recessive genes, both with or without a segregation study to verify biallelism, supported by family history (including the sporadic cases).

### Statistical analysis

The distribution of genotypes was analyzed by chi-square test; Mann–Whitney-U test was used to compare pairs of medians (MedCalc Software, Mariakerke, Belgium). Statistical significance was set at *p* < 0.05. Quantitative data are reported as median ± interquartile range (IQR, QR3-QR1).

## Results

In this study we report the results of genetic testing on 234 Italian patients, 128 of which affected by MD and 106 by CD/CRD. The median age at diagnosis was 44 (IQR, 55.75–25) years and the median age at onset was 18 (IQR, 36.5–8) years.

Subjects who were candidates for the genetic test showed evidence of typical symptoms of MDs, CDs, and CRD, among which the prevailing symptoms were: loss of central vision, central scotoma, photophobia, dyschromatopsia, and adjustment difficulties in the transition from dark to bright environments. Some patients with advanced CRD also reported night blindness and loss of peripheral vision.

**Figure 1:**
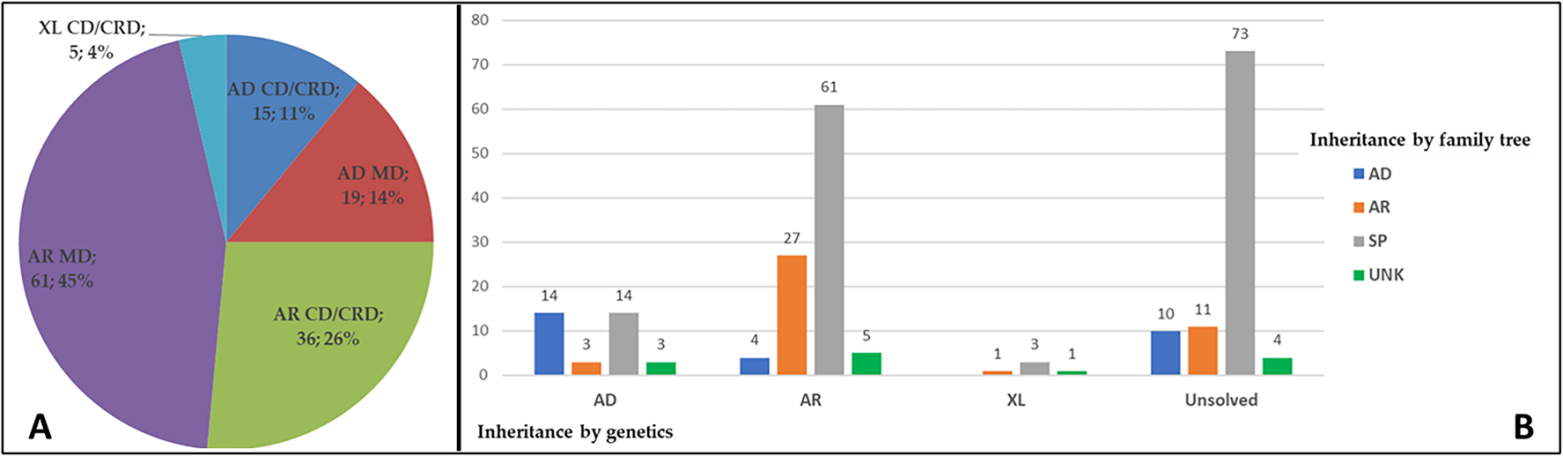
(**A**) Percentage of inheritance patterns based on molecular diagnosis. Data derived from 136 genetically diagnosed patients out of 234 tested individuals. (**B**) Comparison between the inheritance patterns deduced from the pedigrees and those found by the genetic test.

Figure 1A shows that the hereditary pattern was autosomal recessive in 97 probands (71%; 45% MD and 26% CD + CRD), autosomal dominant in 34 (25%; 11% MD and 14% CD + CRD) and X-linked in 5 (4% of CD + CRD).

We traced the inheritance pattern of the 234 probands using the family trees collected during the pre-test genetic counselling sessions. Among them, 151 were sporadic cases (64.5%), while the remaining had a family history that suggested autosomal recessive (42 patients, 17.9%) or autosomal dominant (28 patients, 11.9%) inheritance. For the remaining 13 patients it was either not possible to collect evidence concerning their family history or it was difficult to interpret their pedigrees. Apparently, X-linked MDs, CDs, or CRDs were absent (Fig. 1B). The inheritance pattern was consistent with genetic test inheritance in 27/42 (64.3%) autosomal recessive and 14/28 (50%) autosomal dominant patients, respectively. Sporadic patients solved by genetic testing were 78/151 (51.6%); among them 61/78 (78.2%) were autosomal recessive, 14/78 (17.9%) were autosomal dominant and 3 (3.8%) were X-linked.

The remaining 17 solved patients among whom the inheritance model was determined exclusively by genetic testing were autosomal recessive for 9 out of a total of 97 (9.3%), autosomal dominant for 6 out of a total of 34 (17.6%) and X-linked for 2 out of a total of 5 (40%).

Genetic testing revealed potentially disease-associated variants in 136 patients (58.1% of all recruited patients). Among them, 80 were affected by MD, 28 by CD (two of them presented with cone dystrophy were found to be affected by achromatopsia with pathogenic variants in *CNGB3*, a gene included in the cone dystrophy panel) and 28 from CRD.

Genetic testing was considered inconclusive in 69 (29.5%) probands and uninformative in 29 (12.4%). Probands with inconclusive and with uninformative tests were considered “unsolved cases”, which accounted for 41.9% of all recruited patients (Table 1).

**Table 1:** Numbers and percentages of 234 Italian patients affected by MDs, CDs, and CRDs.

Inconclusive	Uninformative	1st level test	2nd level test
69/98 (70.4%)	29/98 (29.6%)	122/234 (52.1%)	14/41 (34.1%)

Unsolved	Diagnosed		
98/234 (41.9%)	136/234 (58.1%)		

*1st level test includes Sanger, NGS targeted sequencing and MLPA.

The main cause of both MD and CD/CRD cases (59%) was the presence of variants in the *ABCA4* gene, followed by variants in *PRPH2* (9%) and in *BEST1* (6%).

**Figure 2:**
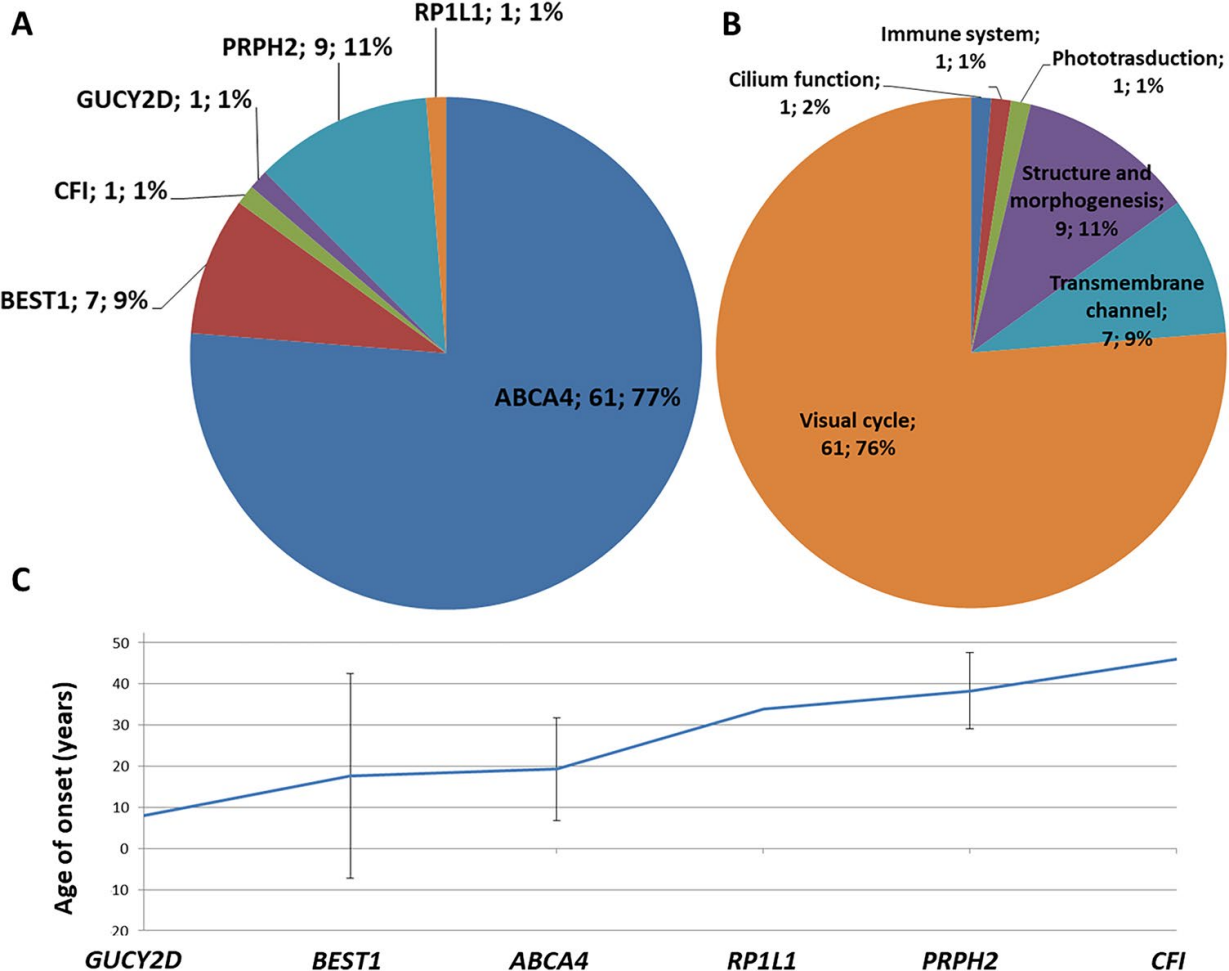
(**A**) Prevalence of gene variants in 80 MD patients. (**B**) Functional classification of variants identified in MD patients. (**C**) Mean age of onset for each gene was calculated on 131 genetically solved subjects (probands and affected relatives). Bars indicate ± SD.

**Figure 3:**
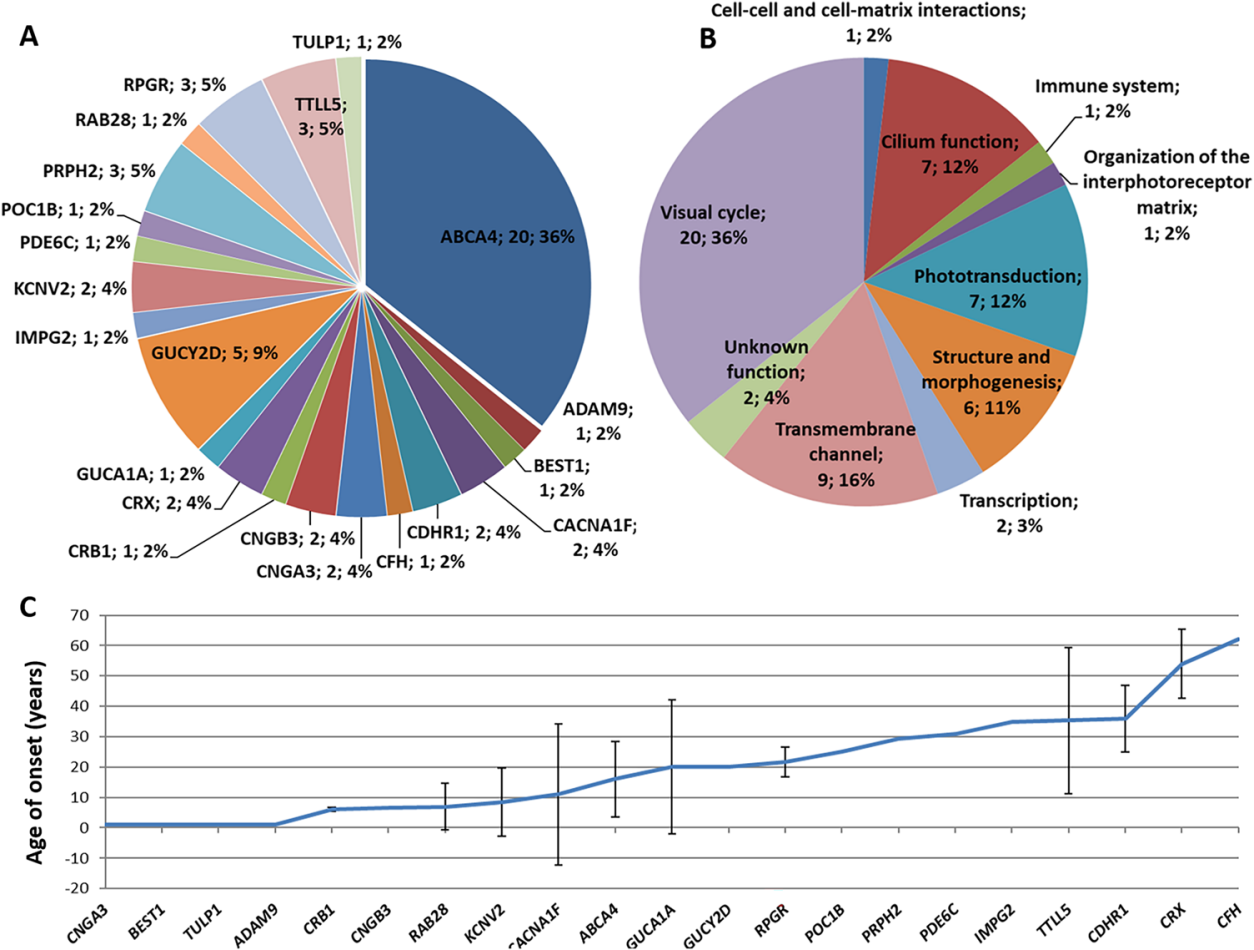
(**A**) Prevalence of gene variants in 56 CD/CRD patients. (**B**) Functional classification of variants identified in CD/CRD patients. (**C**) Mean age of onset for each gene was calculated on 58 genetically solved subjects (probands and affected relatives). Bars indicate ± SD.

The vast majority (77%) of MD cases were caused by homozygous or double heterozygous *ABCA4* variants, followed by heterozygous *PRPH2* (11%) and *BEST1* (9%) variants (Fig. 2A). *ABCA4* was the major gene also in CD/CRD cases (35%), followed by *GUCY2D* (9%), *PRPH2* (5%), and *TTLL5* (5%) (Fig. 3A). The most frequent variant in the whole population of MD and CD/CRD cases was the *ABCA4* c.5882G > A, p.(Gly1961Glu), which recurred in 49 patients (34 MD and 5 CD/CRD), i.e. in 10.5% of all the screened alleles.

The detailed results of genetic tests in MD and CD/CRD patients are listed in Table S2 and Table S3, respectively.

**Figure 4:**
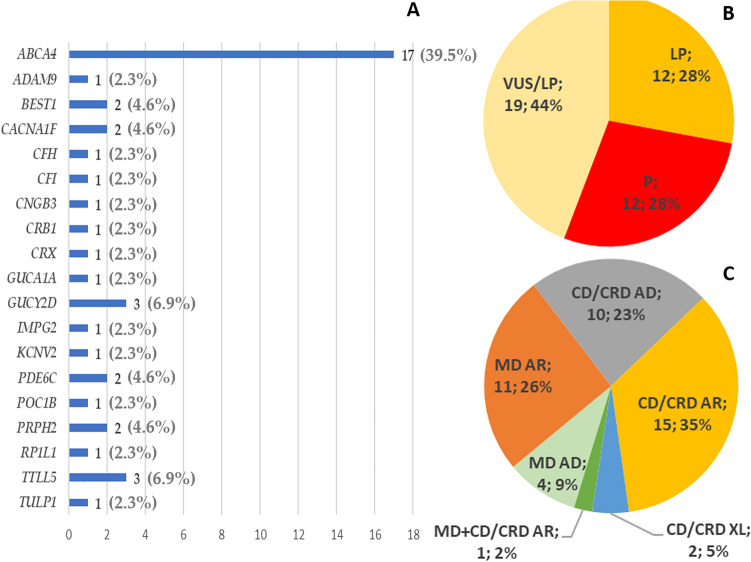
Overview of the 43 unpublished variants found in 44 unrelated patients: (**A**) distribution in genes; (**B**) classification of pathogenicity; (**C**) distribution by clinical phenotype (MD and CD/CRD) and by inheritance pattern (autosomal dominant, autosomal recessive and X-linked).

Overall, we identified 236 different variants (Table 2), 43 of which were unpublished at the time of this report (Fig. 4 and Table 3). These 43 unpublished variants were found in 44 unrelated patients. The *ABCA4* c.5959_5964delinsTG, p.(Gly1987*) variant was indeed found in two unrelated patients, more specifically in patient MD-45 who also carried the c.5882G > A, p.(Gly1961Glu), and patient CD/CRD-20 who carried the c.5714 + 5G > A, p.(Glu1863Leufs*33) (Table S2 and Table S3).

**Table 2:** Genetic variants found in 136 out of 234 tested individuals, classified by type and allelic state.

Variant type	Hemizygous	Heterozygous	Homozygous	Total
Large deletions/insertions		2		2
Missense		153	9	162
Nonsense		20	1	21
Small deletions/insertions	4	24	4	32
Splicing	1	18		19
Total	**5**	**217**	**14**	**236**

**Table 3:** List of the 43 new variants identified in this study.

Disease	Gene	Transmission	RefSeq	Exon/Intron	Nucleotide change	Amino acid change	Allele state	dbSNP rs	VarSome
CD	*ABCA4*	Aut. Recessive	NM_000350	ex1-5	c.1-?_571-?del	p.(?)	HET	NA	P
CD	*ABCA4*	Aut. Recessive	NM_000350	ex8	c.1085_1086del	p.(Tyr362*)	HET	NA	P
CD	*ABCA4*	Aut. Recessive	NM_000350	ex17	c.2624 T > C	p.(Leu875Pro)	HET	NA	VUS/LP
MD	*ABCA4*	Aut. Recessive	NM_000350	ex19	c.2780C > T	p.(Pro927Leu)	HET	NA	VUS/LP
MD	*ABCA4*	Aut. Recessive	NM_000350	ex19	c.2875A > G	p.(Thr959Ala)	HET	rs368846708	LP
MD	*ABCA4*	Aut. Recessive	NM_000350	ex21	c.3167A > T	p.(Asn1056Ile)	HET	NA	LP
CRD	*ABCA4*	Aut. Recessive	NM_000350	ex23	c.(3192-90_3418)_(3418_3562)dup	p.(?)	HET	NA	P
MD	*ABCA4*	Aut. Recessive	NM_000350	ex22	c.3289A > G	p.(Arg1097Gly)	HET	NA	LP
MD	*ABCA4*	Aut. Recessive	NM_000350	ex27	c.3999_4000insACCCCAGAGCCAGAGTGCCAGCCT	p.(Pro1333_Pro1334insThrProGluProGluCysGlnPro)	HET	NA	LP
MD	*ABCA4*	Aut. Recessive	NM_000350	ex27	c.4085G > T	p.(Arg1362Ile)	HET	NA	VUS/LP
MD	*ABCA4*	Aut. Recessive	NM_000350	ex28	c.4217del	p.(His1406Profs*29)	HET	NA	P
MD	*ABCA4*	Aut. Recessive	NM_000350	ex33	c.4734_4739delinsCC	p.(Phe1579Glnfs*8)	HET	NA	P
MD	*ABCA4*	Aut. Recessive	NM_000350	ex38	c.5384 T > G	p.(Leu1795*)	HET	NA	P
MD	*ABCA4*	Aut. Recessive	NM_000350	ex43	c.5910_5912dup	p.(Leu1971dup)	HET	NA	LP
MD + CD	*ABCA4*	Aut. Recessive	NM_000350	ex43	c.5959_5964delinsTG	p.(Gly1987*)	HET	NA	P
CD	*ABCA4*	Aut. Recessive	NM_000350	ex43	c.5959G > T	p.(Gly1987Tpr)	HET	NA	LP
MD	*ABCA4*	Aut. Recessive	NM_000350	ex45	c.6184_6188del	p.(Val2062Argfs*33)	HET	NA	P
CRD	*ADAM9*	Aut. Recessive	NM_003816	ex8	c.725 T > G	p.Leu242Arg	HOM	NA	VUS/LP
CRD	*BEST1*	Aut. Dominant	NM_004183	ex4	c.318dup	p.(Met107Hisfs*125)	HET	NA	P
MD	*BEST1*	Aut. Dominant	NM_004183	ex7	c.718_720dup	p.(Val240dup)	HET	NA	LP
CRD	*CACNA1F*	X-linked	NM_005183	ex23	c.2804_2806del	p.(Phe935del)	HEM	rs782068089	VUS/LP
CRD	*CACNA1F*	X-linked	NM_005183	int23	c.2874-1G > C	p.(?)	HEM	NA	P
CRD	*CFH*	Aut. Dominant	NM_000186	ex16	c.2440C > T	p.(Pro814Ser)	HET	NA	VUS/LP
MD	*CFI*	Aut. Dominant	NM_000204	ex13	c.1573T > C	p.(Ser525Pro)	HET	NA	VUS/LP
CD	*CNGB3*	Aut. Recessive	NM_019098	ex2	c.143del	p.(Gly48Valfs*35)	HOM	NA	LP
CRD	*CRB1*	Aut. Dominant	NM_201253	ex7	c.2149G > T	p.(Gly717Cys)	HET	NA	LP
CRD	*CRX*	Aut. Dominant	NM_000554	ex4	c.329del	p.(Gly110Alafs*77)	HET	rs761108522	P
CRD	*GUCA1A*	Aut. Dominant	NM_000409	ex4	c.312_313delinsGC	p.(Asn104_Gly105delinsLysArg)	HET	NA	VUS/LP
CD	*GUCY2D*	Aut. Dominant	NM_000180	ex2	c.286T > C	p.(Phe96Leu)	HET	NA	VUS/LP
MD	*GUCY2D*	Aut. Dominant	NM_000180	ex13	c.2480A > C	p.(Tyr827Ser)	HET	NA	VUS/LP
CD	*GUCY2D*	Aut. Dominant	NM_000180	ex13	c.2546C > G	p.(Thr849Arg)	HET	NA	LP
CRD	*IMPG2*	Aut. Dominant	NM_016247	ex2	c.283G > C	p.(Glu95Gln)	HET	rs1198094357	VUS/LP
CD	*KCNV2*	Aut. Recessive	NM_133497	ex2	c.1427T > G	p.(Leu476Arg)	HOM	rs796658305	LP
CD	*PDE6C*	Aut. Recessive	NM_006204	ex17	c.2087C > T	p.(Thr696Met)	HET	rs41290222	VUS/LP
CD	*PDE6C*	Aut. Recessive	NM_006204	ex20	c.2367 + 1_2367 + 5del	p.(?)	HET	rs796051871	P
CRD	*POC1B*	Aut. Recessive	NM_172240	ex6	c.587C > T	p.(Pro196Leu)	HET	NA	VUS/LP
CD	*PRPH2*	Aut. Dominant	NM_000322	ex1	c.568A > G	p.(Lys190Glu)	HET	NA	VUS/LP
CD	*PRPH2*	Aut. Dominant	NM_000322	ex2	c.621C > A	p.(Asp207Glu)	HET	NA	LP
MD	*RP1L1*	Aut. Dominant	NM_178857	ex2	c.563 T > C	p.(Leu188Pro)	HET	NA	VUS/LP
CRD	*TTLL5*	Aut. Recessive	NM_015072	ex10	c.800T > C	p.(Leu267Pro)	HOM	NA	VUS/LP
CRD	*TTLL5*	Aut. Recessive	NM_015072	ex13	c.1060G > A	p.(Val354Met)	HOM	rs781509883	VUS/LP
CRD	*TTLL5*	Aut. Recessive	NM_015072	ex17	c.1442G > C	p.(Gly481Ala)	HOM	rs771482604	VUS/LP
CRD	*TULP1*	Aut. Recessive	NM_003322	ex8	c.822G > T	p.(Lys274Asn)	HET	NA	VUS/LP

All variants are classified for pathogenicity, according to the American College of Medical Genetics and Genomics guidelines. Data derived from 44 unrelated patients.

ex, exon; int, intron; HET, heterozygous; HOM, homozygous; HEM, hemizygous; P, pathogenic; LP, likely pathogenic; VUS, variant of unknown significance.

It should be noted that 15 probands (10 MD and 5 CD/CRD) were found to harbor > 2 *ABCA4* variants, and in 3 of them 2 variants were *in cis* configuration, as confirmed by the family segregation study: patients MD-8 and MD-31, affected by MD (Table S2), and patient CD/CRD-10, affected by CD/CRD (Table S3). The contribution to the pathogenicity of *in cis* variants is difficult to interpret and cannot be solved without further experimental evidence. Among the others, patients MD-16 and CD/CRD-3 can be suspected to have the same *in cis* variants of CD/CRD-10, namely the *ABCA4* p.(Leu541Pro) and p.(Ala1038Val). In particular, these *in cis* variants were found *in trans* with the c.1239 + 1G > C in patient CD/CRD-10, as verified by the segregation study performed on the proband and on his healthy parents. The same variants were found in patient MD-16 and in his affected sister, both carriers of a third variant, the p.(Arg1898Cys). Patient CD/CRD-3 also carried a third variant, the p.(Arg508Cys), but it was impossible to extend the study to other family members. For these reasons, we cannot confirm the allelic configuration in MD-16 and CD/CRD-3 patients until further family segregation studies are performed.

### Genotype–phenotype correlations

The family segregation study was useful in order to reach a potential genetic diagnosis in 32 out of 41 (78%) cases. We noted a genotype–phenotype concordance between affected subjects of the same family carrying the same variant in almost all families, except in four cases. Proband CD/CRD-41 carrying the *IMPG2* variant p.(Glu95Gln), for example, followed for years at the same Institute, initially presented as MD then unpredictably evolved as CRD, while her sister had a milder, more stable phenotype, which slightly involve the macula. Similarly, proband CD/CRD-48 carrying the *PRPH2* p.(Lys190Glu) variant had a clinical picture of CD while her daughter had a less severe picture without macula involvement. This difference could be related to the age difference between the 63-year-old proband and her 41-year-old daughter. On the other hand, proband CD/CRD-13 carrying two missense variants in *ABCA4* presented an extensive form of CRD with very altered ERG of the cones, while her sister presented a clinical picture of non-severe MD and normal ERG. In this case, the ages of onset between the two subjects are also very different, as the disease began at 20 in the proband and at 40 in her sister. In the family of proband MD-76 there were a total of 4 subjects with the variant *PRPH2* p.(Arg172Trp), two young people (27 and 34 years old) with MD at initial stages and two older (54 and 57 years old) with severe MD and later involvement of the cones. Interestingly, the same variant was also found in the proband CD/CRD-47 who showed a clinical picture that is typical of a CRD.

From our case series, the genes found that are known to cause both MDs and CDs/CRDs were *ABCA4*, *BEST1*, *GUCY2D*, and *PRPH2*. The inheritance pattern found in our patients was autosomal recessive for *ABCA4* and autosomal dominant for all the other genes.

Comparing the median age at examination of patients with MD (42 years, IQR 52–23.75) and CD/CRD (47 years, IQR 60–30) no statistically significant difference was found between the two groups (*p* = 0.0733). Also considering only patients with *ABCA4* variants, the differences between the median age of MD (34.5 years, IQR 48–23) and CD/CRD patients (48 years, IQR 64–26.5) was not statistically significant (*p* = 0.0831).

**Figure 5:**
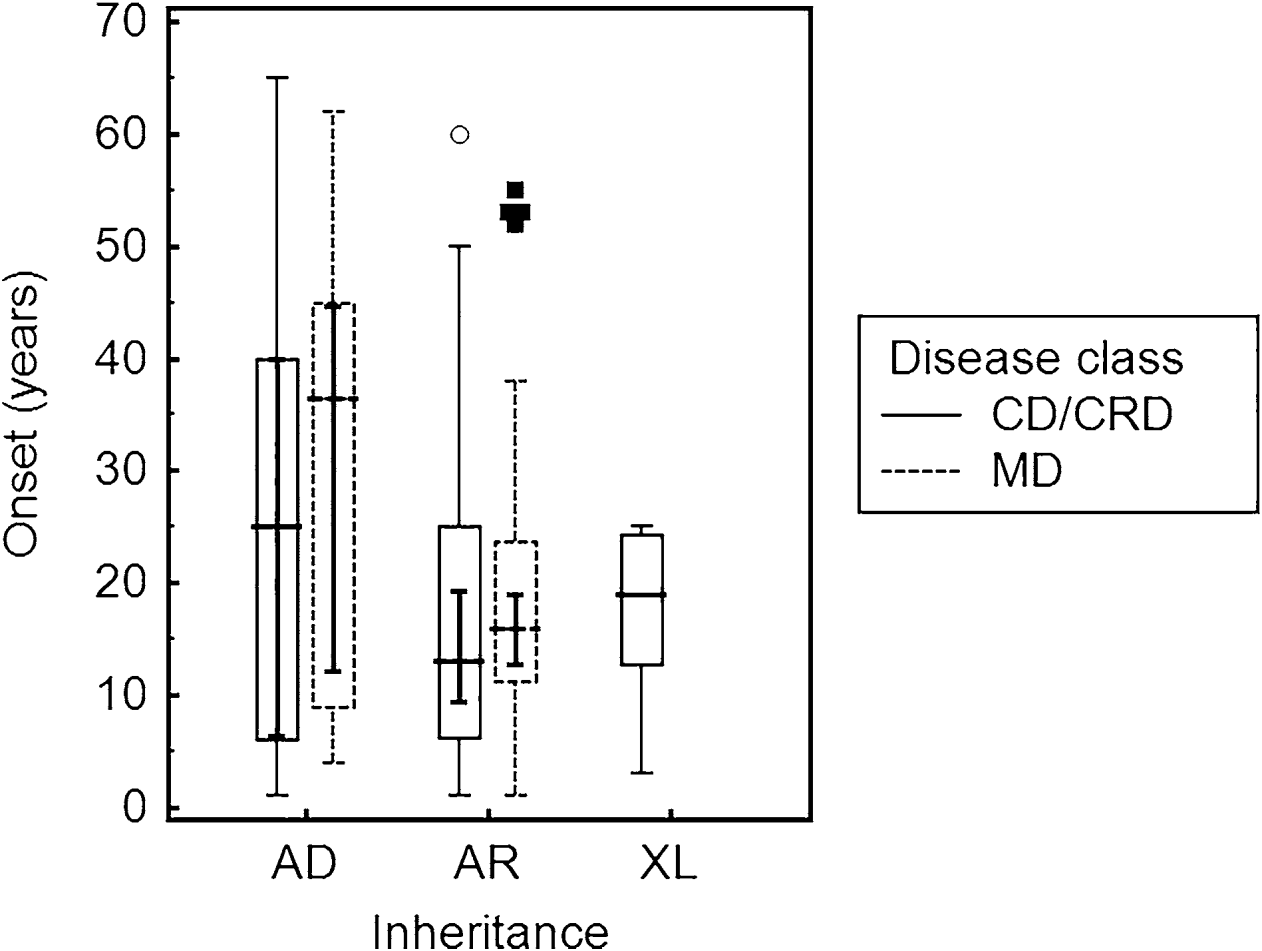
Median age of onset of MD and CD/CRD by inheritance after molecular testing, calculated on 131 genetically solved subjects (both probands and affected relatives). AD-MD 36.5 (IQR, 44.75–11) years; AD-CD/CRD 25 (IQR, 40–6.25) years; AR-MD 16 (IQR, 23.5–11.5) years; AR-CD/CRD 13 (IQR, 25–6.5) years; XL-CD/CRD 19 (IQR, 24–16) years.

The median age at onset was of 17 (IQR, 34–11) years for MD and 15.5 (IQR, 30–6.25) years for CD/CRD (*p* = 0.2437) irrespective of the inheritance pattern, a difference that was not statistically significant; inversely, autosomal dominant patients had a significantly later onset (34.5, IQR 44.5–8) as compared to autosomal recessive patients (15, IQR 24–10) (*p* = 0.0234) regardless of the disease class: statistics is not informative for X-linked patients, due to the low number of patients (only 5) in this category. Figure 5 shows the median age at onset for each disease group, divided by inheritance pattern.

Comparing the median age at onset considering only patients with *ABCA4* variants, the differences between MD and CD/CRD patients (16 years, IQR 24–11.5 and 12 years, IQR 17–8, respectively) was not significant (*p* = 0.0665).

We also compared the median ages of disease onset of MD and CD/CRD patients with two variants in *ABCA4* (15 years, IQR 23–11) with the median ages of onset of patients with only one variant (25 years, IQR 40–11.5) and there was no significant difference (*p* = 0.0505).

We compared the median age of onset of solved MD + CD/CRD patients (17 years, IQR 33–10) versus unsolved cases (21.5 years, IQR 40–6.25) tested with NGS targeted sequencing and found that they did not differ significantly (*p* = 0.5748). The differences remain non-significant even dividing patients by disease class (MD and CD/CRD; data not shown).

Finally, we compared the distribution of truncating and non-truncating *ABCA4* variants among genotypes (maternal/paternal) of MD patients with those affected by CD/CRD and we verified whether these were associated with the age of onset of the disease. The genotype distribution was not statistically different (*p* = 0.9611): it was 43.5% non-truncating/non-truncating, 46.8% non-truncating/truncating and 9.7% truncating/truncating for MD patients and 40% non-truncating/non-truncating, 50% non-truncating/truncating and 10% truncating/truncating for CD/CRD patients.

Due to the low number of MD and CD/CRD patients with biallelic truncating *ABCA4* variants, the median age of disease onset was checked by comparing patients with non-truncating variants versus all those who had at least one truncating variant. When considering MD and CD/CRD patients together, the difference between the median age of disease onset in patients with non-truncating variants (15 years, IQR 19.7–9.2) was not statistically significant as compared to that of patients with truncating variants (14 years, IQR 23.7–11.2) (*p* = 0.5421). For patients with CD/CRD, the median age of disease onset among carriers of non-truncating variants was 13 years (IQR 16–8) while that of patients with truncating variants was 12 years (IQR 19–7.5) (*p* = 0.8148). Similarly, this difference was not statistically significant for MD patients either: the median age of disease onset was 15 years (IQR 21.2–9.7) for patients with non-truncating variants and 17 years (IQR 24–12) for patients with truncating variants (*p* = 0.5259).

## Discussion

In this study, targeted NGS analysis proved to be effective in identifying pathogenetic variants in 136 out of 234 MD or CD/CRD patients (58.1%) and allowed us to discover 43 unpublished variants potentially associated with the diseases. We found pathogenic variants in 23 different genes (Table 4), mostly in *ABCA4* (59% of solved cases, 34.6% of the total cohort). The other most commonly mutated genes were *PRPH2*, *BEST1* and *GUCY2D* (8.7%, 5.8% and 4.3% of solved cases, respectively).

**Table 4:** List of genes encoding proteins involved in the pathogenesis of MDs, CDs, and CRDs in our cohort.

Gene	Diagnosed patients	Percentage	Protein	Functional category
**Macular dystrophies**				
*GUCY2D*	1	1.3%	Retinal-specific guanylate cyclase	Phototransduction
*ABCA4*	61	76%	ATP-binding cassette transporter—retinal	Visual cycle
*PRPH2*	9	11%	Peripherin 2	Structure and morphogenesis
*RP1L1*	1	1.3%	Retinitis pigmentosa 1-like protein 1	Cilium function
*CFI*	1	1.3%	Complement factor I	Immune system
*BEST1*	7	9%	Bestrophin 1	Transmembrane channel
**Cone and cone-rod dystrophies**				
*GUCA1A*	1	12%	Guanylyl cyclase-activating protein 1	Phototransduction
*GUCY2D*	5		Retinal-specific guanylate cyclase	
*PDE6C*	1		cGMP-specific cone phosphodiesterase 6C alpha prime protein	
*ABCA4*	20	35%	ATP-binding cassette transporter—retinal	Visual cycle
*PRPH2*	3	12%	Peripherin 2	Structure and morphogenesis
*CDHR1*	2		Cadherin-related family member 1 (protocadherin 21)	
*CRB1*	1		Protein crumbs homolog 1	
*RPGR*	3	12%	Retinitis pigmentosa GTPase regulator	Cilium function
*TTLL5*	3		Tubulin tyrosine ligase-like family member 5	
*POC1B*	1		POC1 (proteome of centriole 1) centriolar protein B	
*BEST1*	1	16%	Bestrophin 1	Transmembrane channel
*CACNA1F*	2		L-type voltage-gated calcium channel alpha-1 subunit	
*CNGA3*	2		Cone photoreceptor cGMP-gated cation channel alpha subunit	
*CNGB3*	2		Cone cyclic nucleotide-gated cation channel beta 3 subunit	
*KCNV2*	2		Potassium channel subfamily V member 2	
*CRX*	2	3%	Cone-rod otx-like photoreceptor homeobox transcription factor	Transcription
*ADAM9*	1	2%	ADAM metallopeptidase domain 9 (meltrin gamma) protein	Cell–cell and cell–matrix interactions
*CFH*	1	2%	Complement factor H	Immune system
*IMPG2*	1	2%	Interphotoreceptor matrix proteoglycan 2	Organization of the interphotoreceptor matrix
*RAB28*	1	4%	RAB28 member of RAS oncogene family	Unknown function
*TULP1*	1		Tubby-like protein 1	

All the molecular changes in these proteins lead to dysfunctions in different visual processes.

Our results highlight the genetic and phenotypic heterogeneity of MDs and CDs/CRDs as already shown by similar studies performed in other populations^25–27^. Compared with the results reported by Birtel et al.^25^ on 251 consecutive German patients with a smaller panel comprising 48 genes, our detection rate has been somewhat lower (74% in the aforementioned study vs 58.1% in our cohort), but these Authors found that variants in the same genes (*ABCA4*, *PRPH2* and *BEST1*) accounted for most of the diagnosed patients (57% vs. 74% in our cohort). The difference in detection rate could be related to differences in the population characteristics, such as the age of onset of the disease. Indeed some authors suggested that the diagnostic yield of targeted NGS increases in patients with early onset of the disease as compared to those with later onset, probably because the latter may have a multigenic or multifactorial etiology that includes accumulated environmental factors^26^. However, this is not the case, since our patients showed a significantly earlier age of onset (17, IQR 31.5–9.5) than the German cohort (35, IQR 59–18) (*p* = 0.0001). Furthermore, in our cohort the median age of onset of MDs + CDs/CRDs in patients solved by the NGS Targeted Sequencing test did not differ significantly from that of unsolved cases. Comparing their findings with similar studies, Birtel et al.^25^ suggested that some discrepancies may be related to cohort size and ethnicity, variable inclusion criteria, and the efficiency of molecular genetic testing and data analysis; we agree with this interpretation. The genetic heterogeneity of MD and CD/CRD has been replicated in all other studies e.g. in a French cohort of 96 CD/CRD patients screened with an NGS panel of 123 genes, Boulanger-Scemama et al.^27^ found likely causative variants in 62% of cases with the *ABCA4* gene being again the most commonly affected (25.4%, 15 out of 59 solved cases) followed by *GUCY2D* (8.5%, 5/59), *CRX* and *PROM1* (6.7%, 4/59 cases each). Interestingly, variants in *ABCA4* were far less frequent in the Chinese cohort reported by Huang et al.^28^ who reported on NGS screening of 163 patients with cone-rod dystrophy and achieved a 57% detection rate; in their cohort the most frequently mutated gene was *CNGA3* (57%, 53/93 solved patients) and *ABCA4* variants were identified only in 6/93 (6.5%) patients. The two-step diagnostic flow-chart of the Chinese study (first analysis of all CRD genes and then whole exome sequencing targeting all the genes that were known to be responsible for other forms of retinal degeneration) is not a very different approach from the one adopted in our study. Instead, there is an important demographic difference between the two populations in comparison; the Chinese study focused on a pediatric population and the *CNGA3* gene is known to be associated with early-onset IRDs^29^. However, comparing their results with available literature data concerning other populations, Huang et al. suggested in their study that the observed differences might in part be due to ethnic differences.

In a cohort of 43 Japanese CD/CRD patients analyzed using an NGS exome-sequencing panel targeting 193 known inherited eye disease genes, genetic diagnoses were made in 12 (27.9%) patients, and *ABCA4* was the most representative mutated gene (9.3%, 4/43 of the total cohort; 33.3%, 4/12 of solved cases)^30^. The detection rate was much lower than that of our CD/CRD population (52.8%, 56/106 solved patients), a result that had already been described by the same authors in comparison with another European cohort (the French cohort mentioned above)^27,30^.

In the study of Kim et al. were reported the results of an NGS strategy that considered a panel of 204 known pathogenic genes associated with IRDs for the genetic screening of 86 clinically diagnosed Korean IRD patients^31^. Molecular diagnoses were made in 38/86 (44.2%) IRD patients with *ABCA4* representing the most frequently mutated gene (9.3%, 8/86 of the total cohort; 21%, 8/38 of solved cases).

In both the Japanese and Korean cohort papers, it was reported that detailed family history collection was obtained to determine the presumed inheritance traits. Unfortunately, these data were not available for comparison with our results.

All the differences above reported underline the genetic heterogeneity of MDs and CDs/CRDs among world populations and suggest that NGS panels encompassing an ever-growing number of causative genes will be needed in order to detect the genetic determinants of most patients and allow precise genetic counselling to their relatives.

In our study, *ABCA4* variants, both in homozygous or compound heterozygous state, were by far the most frequent cause of MDs or CDs/CRDs. Since *ABCA4* is the major gene for both diseases, these could be classified as *ABCA4*-retinopathies.

Although *ABCA4* heterozygous variants have been widely reported in association with increased risk of age-related macular degeneration 2 (ARMD2, OMIM # 153800) in the literature^32^, and these patients are known to develop central retinal degeneration in older age, patients bearing monoallelic *ABCA4* variants have been considered as “unsolved”. Indeed, the median age of onset of our patients with monoallelic variants was 25 (IQR, 40–11.5) years. Therefore we cannot exclude that these patients with monoallelic variants include some unconfirmed *ABCA4*-retinopathy (due to lack of deep *ABCA4* sequencing or MLPA analysis), possibly some ARMD, or even that the genetic cause is to be found elsewhere.

As previously mentioned, when their first genetic tests were unsolved, patients underwent second level NGS analyses for non-syndromic Inherited Retinal Dystrophies, using a panel with 138 genes. This test revealed causative pathogenetic variants in a high percentage of cases (34.1%, Table 1), allowing us to confirm the diagnosis of inherited retinal dystrophy. The cases that remained unsolved after this second level panel may be considered candidates for Whole Exome Sequencing. However, it should be pointed out that patients with phenotypes within the spectrum of *ABCA4*-retinopathies and one variant in *ABCA4* would first benefit from whole *ABCA4* gene sequencing in search of known deep-intronic variants, before exome sequencing. Indeed, such variants were indicated as an important cause of disease^33^. Unfortunately, these have not been assessed here thus representing a limitation of our study and a possible reason for not determining the molecular cause of disease in these patients.

*ABCA4*, encoding a flippase involved in the visual cycle, plays the major role in causing MD (61/80) in our Italian patients (Table 4 and Fig. 2), despite being also the most frequent cause also of CD/CRD (Table 4 and Fig. 3). Figures 2B and 3B show the many other functions of proteins encoded by the 24 causative genes that we identified, including the visual cycle, phototransduction and ciliary function. Overall, we found 43 variants that were unpublished at the time of this report (Fig. 4 and Table 3). While the functions of most of these genes are known, the effects of these specific variants on the function of the corresponding proteins are still to be established.

As far as we know, this is the first report analyzing the genetic characteristics of a large Italian cohort of patients with CD/CRDs, since the already published studies on Italian patients with MD were performed over 10 years ago and were mainly limited to Stargardt Disease^34,35^.

Thanks to our targeted NGS approach based on an extensive panel of causative genes, we achieved a satisfactory detection rate of 58.1%; however, considering the large number of variants identified and the differences in each patient’s clinical history, we realized that many challenges still lay ahead. Although our diagnostic ability has increased, we are still unable to predict the severity and natural history of an IRD only based on the knowledge of the underlying genetic variant(s). The variability of clinical features among patients belonging to the same family and sharing the same variants are sometimes remarkable and must depend on a number of other genetic and environmental determinants. Understanding the dynamics of allelic and locus heterogeneity^36^, while also considering the contribution of mutation load in other relevant genes^37–40^, is crucial to study complex phenomena, such as variable expressivity and reduced penetrance of IRDs.

An accurate molecular diagnosis can help predict a prognosis, especially when genes that cause CRDs are detected in MD patients. Among them, for example, *GUCY2D* has a more severe prognosis than other genes associated with MDs. In our work, we were unable to demonstrate differences between the *ABCA4* genotypes that caused CDs/CRDs from those that caused MD. However, *ABCA4* is known to exhibit a great variability of phenotypic expressions, which may also depend on other genetic factors, apart from the degree of pathogenicity of the genetic variants predicted by the in silico software or the type of mutation. Genetic variants can also be distributed differently among countries and different ethnic groups and thus influence the pathological phenotype differently; for example, it has been found to be milder among Africans than among Europeans^41,42^. This information is important for patient counselling about prognosis.

Furthermore, obtaining the genetic definition of a disease is mandatory to enter clinical trials as targeted treatment options will be available to patients in the near future. There are several gene therapy-based treatment options for *ABCA4*-distrophies that may become available^41,43^, while gene therapies for *GUCY2D*^44^ and *RPGR*^45^ were shown to have greatly promising effects in humans.

The goal of personalized medicine is to tailor the appropriate therapy for the individual patient and this may require an understanding the pathogenic significance of thousands of genetic variants, performing family segregation studies and implementing functional tests for these same variants. Furthermore, extensive and detailed knowledge of the molecular interactions (interactome) taking place in the human retina will guide the development of new and more powerful NGS panels. Ideally, personalized medicine should be able to predict the patients’ disease severity and evolution based on a set of individual genetic and metabolic parameters. We believe that the detailed study of genetic determinants is the prerequisite for the development of effective preventive measures as well as future therapies for IRD patients.

## Supplementary Information

Supplementary Information.

## Data Availability

Clinical data reported in this work are available upon request from the corresponding author.
