# Supplementary material for: Genetic characteristics of 234 Italian patients with macular and cone/cone-rod dystrophy
Source: Sci Rep. 2022 Mar 8;12:3774. doi: 10.1038/s41598-022-07618-1 (PMC8904500; doi:10.1038/s41598-022-07618-1)
Supplement: Supplementary file 1 — Supplementary Information. [file 41598_2022_7618_MOESM1_ESM.pdf]

Genetic Characteristics of 234 Italian Patients with Macular and Cone/Cone-Rod Dystrophy.

Benedetto Falsini[1,8], Giorgio Placidi[1,8], Elisa De Siena[1], Pietro Chiurazzi[2], Angelo Maria Minnella[1], Maria Cristina Savastano[1], Lucia Ziccardi[3], Vincenzo Parisi[3], Giancarlo Iarossi[4], Marcella Percio[6], Barbora Piteková[5], Giuseppe Marceddu[7], Paolo Enrico Maltese[6,9], Matteo Bertelli[6,7,9]

[1]Ophthalmology Unit, Fondazione Policlinico Universitario "A. Gemelli" IRCCS/Universita' Cattolica del S. Cuore, Rome, Italy;
[2]UOC Genetica Medica, Fondazione Policlinico Universitario "A. Gemelli" IRCCS & Istituto di Medicina Genomica, Universita' Cattolica del S. Cuore, Rome, Italy;
[3]IRCCS Fondazione Bietti, Rome, Italy;
[4]Ophthalmology Unit, Bambino Gesu' Children's Hospital, IRCSS, Rome, Italy;
[5]Department of Pediatrics, National Institute of Children's Diseases, Faculty of Medicine, Comenius University Bratislava, Slovak Republic;
[6]MAGI'S Lab s.r.l., Rovereto, Italy;
[7]MAGI Euregio s.c.s., Bolzano, Italy;
[8]These authors contributed equally: Benedetto Falsini and Giorgio Placidi
[9]These authors jointly supervised this work: Paolo Enrico Maltese and Matteo Bertelli

Address for correspondence
Dr. Paolo Enrico Maltese
Magi's Lab s.r.l., Via Delle Maioliche 57/D
38068 Rovereto (TN), Italy
Tel.: 39-0464 662771
Fax: 39-0464 425634
Email: paolo.maltese@assomagi.org

Table S1. Genes included in custom NGS targeted sequencing panels.

NON-SYNDROMIC IRDs		CONE DYSTROPHY		MACULAR DYSTROPHY			STARGARDT DISEASE	
PANEL NAME (PERIOD OF USE)		PANEL NAME (PERIOD OF USE)	PANEL NAME (PERIOD OF USE)	PANEL NAME (PERIOD OF USE)	PANEL NAME (PERIOD OF USE)	PANEL NAME (PERIOD OF USE)	PANEL NAME (PERIOD OF USE)	PANEL NAME (PERIOD OF USE)
OCULAR (2017-2019)		OSS_NEW (2014-2018)	OCULAR (2017-2019)	OSS_NEW MD_Rev 0 (2014-2017)	OSS_NEW MD_Rev 1 (2017-2018)	OCULAR (2017-2019)	OSS_NEW (2014-2018)	OCULAR (2017-2019)
ABCA4	LCA5	CNGA3	ABCA4	ABCA4	ABCA4	ABCA4	ABCA4	ABCA4
ABCB6	LRAT	RAB28	ADAM9	BEST1	BEST1	BEST1	ELOVL4	CNGB3
ADAM9	LRIT3	GUCY2D	AIPL1	EFEMP1	C8orf37	C8orf37	PROM1	ELOVL4
AGBL5	MAK	PROM1	C8orf37	ELOVL4	CRX	CDH3	PRPH2	PROM1
AHI1	MERTK	GUCA1A	CACNA1F	IMPG1	EFEMP1	CFH		PRPH2
AIPL1	NEK2	CERKL	CACNA2D4	IMPG2	ELOVL4	CFI		
ARHGEF18	NMNAT1	CACNA1F	CDHR1	PROM1	GUCA1A	CRX		
ARL2BP	NR2E3	SEMA4A	CEP78	PRPH2	IMPG1	DRAM2		
ARL6	NRL	CRX	CFAP410	RP1L1	IMPG2	EFEMP1		
ATF6	NYX	AIPL1	CNGA3	RPGR	PROM1	ELOVL4		
BBS2	OFD1	RPGRIP1	CRX	TIMP3	PRPH2	GUCA1A		
BEST1	OPA1	ABCA4	DRAM2		RAX2	IMPG1		
C1QTNF5	OPA3	PITPNM3	GUCA1A		RIMS1	IMPG2		
C8orf37	OTX2	PRPH2	GUCY2D		RLBP1	PRDM13		
CA4	PCARE	CNGB3	IFT81		RP1L1	PROM1		
CABP4	PDE6A	ADAM9	KCNV2		RPGR	PRPH2		
CACNA1F	PDE6B	RPGR	PDE6C		RPGR	RAX2		
CACNA2D4	PDE6C	CDHR1	PITPNM3		SEMA4A	RIMS1		
CDH3	PDE6G	RIMS1	POC1B		TIMP3	RLBP1		
CDHR1	PDE6H	RAX2	PROM1		TTC8	RP1L1		
CEP290	PITPNM3		PRPH2			RPGR		
CEP78	POC1B		RAB28			RPGR		
CERKL	POMGNT1		RAX2			SEMA4A		
CFAP410	PRCD		RIMS1			TIMP3		
CFH	PROM1		RPGR			TTC8		
CFI	PRPF3		RPGR					
CHM	PRPF31		RPGRIP1					
CLN3	PRPF4		SEMA4A					
CLRN1	PRPF6		TTLL5					
CNGA1	PRPF8		UNC119					
CNGA3	PRPH2							
CNGB1	RAB28							
CNGB3	RAX2							
CRB1	RBP3							
CRX	RD3							
CTNNA1	RDH12							
DHDDS	RDH5							
DNM1L	REEP6							
DRAM2	RGR							
EFEMP1	RHO							
ELOVL4	RIMS1							
EYS	RLBP1							
FAM161A	ROM1							
FLVCR1	RP1							
FSCN2	RP1L1							
GDF6	RP2							
GNAT1	RP9							
GNAT2	RPE65							
GNB3	RPGR							
GPR179	RPGRIP1							
GRK1	RS1							
GRM6	SAG							
GUCA1A	SEMA4A							
GUCA1B	SLC24A1							
GUCY2D	SLC7A14							
HGSNAT	SNRNP200							
IDH3A	SPATA7							
IDH3B	TIMP3							
IFT140	TMEM126A							
IFT172	TOPORS							
IFT81	TRPM1							
IMPDH1	TTC8							
IMPG1	TTLL5							
IMPG2	TUBB4B							
IQCB1	TULP1							
KCNJ13	UNC119							
KCNV2	USH2A							
KIZ	ZNF408							
KLHL7	ZNF513							

Table S2. Demographic and genetic characteristics of MD probands.

ID	Sex	Age	ONSET	Gene	RefSeq	Exon/ intron	Nucleotide change	Amino acid change	Allele state	dbSNP rs	VARSOME	Transmission	REF
MD-1	F	31	25	*ABCA4*<br>*ABCA4*	NM_000350	ex34<br>ex40	c.4793C>A<br>c.5603A>T	p.(Ala1598Asp)<br>p.(Asn1868Ile)	HET<br>HOM	rs61750155<br>rs1801466	P<br>VUS/LP	AR	1<br>2
MD-2	F	33	24	*ABCA4*<br>*ABCA4*	NM_000350	ex33<br>ex42	c.4710del<br>c.5882G>A	p.(Ile1571Serfs*10)<br>p.(Gly1961Glu)	HET<br>HET	rs281865381<br>rs1800553	LP<br>P	AR	3<br>4
MD-3	F	21	8	*ABCA4*	NM_000350	ex43	c.5917del	p.(Val1973*)	HOM	rs61751389	P	AR	5
MD-4	F	46	17	*ABCA4*<br>*ABCA4*	NM_000350	ex47<br>ex42	c.6445C>T<br>c.5882G>A	p.(Arg2149*)<br>p.(Gly1961Glu)	HET<br>HET	rs61750654<br>rs1800553	P<br>P	AR	6<br>4
MD-5	F	74	25	*ABCA4*<br>*ABCA4*	NM_000350	ex30<br>ex46	c.4354G>T<br>c.6316C>T	p.(Glu1452*)<br>p.(Arg2106Cys)	HET<br>HET	rs886044742<br>rs61750648	P<br>P	AR	7<br>4
MD-6	F	16	10	*ABCA4*<br>*ABCA4*<br>*ABCA4*	NM_000350	ex34<br>ex42<br>ex3	c.4775G>A<br>c.5882G>A<br>c.288C>A	p.(Gly1592Asp)<br>p.(Gly1961Glu)<br>p.(Asn96Lys)	HET<br>HET<br>HET	rs1425552175<br>rs1800553<br>rs886039297	P<br>P<br>LP	AR	8<br>4<br>9
MD-7	M	34	23	*ABCA4*	NM_000350	ex43	c.5917del	p.(Val1973*)	HOM	rs61751389	P	AR	5
**MD-8**	F	45	NA	*ABCA4*<br>*ABCA4*	NM_000350	ex6<br>ex24<br>ex42	*c.667A>C*<br>*c.3607G>A*<br>c.5882G>A	*p.(Lys223Gln)*<br>*p.(Gly1203Arg)*<br>p.(Gly1961Glu)	HET<br>HET<br>HET	rs147619585<br>rs1064793011<br>rs1800553	VUS/LP<br>P<br>P	AR	9<br>9<br>4
**MD-9**	M	20	NA	*ABCA4*<br>*ABCA4*	NM_000350	ex15<br>ex42	c.2300T>A<br>c.5882G>A	p.(Val767Asp)<br>p.(Gly1961Glu)	HET<br>HET	rs61751395<br>rs1800553	P<br>P	AR	10<br>4
MD-10	M	43	20	*ABCA4*<br>*ABCA4*	NM_000350	ex1<br>ex42	c.61C>T<br>c.5882G>A	p.(Gln21*)<br>p.(Gly1961Glu)	HET<br>HET	rs770272033<br>rs1800553	P<br>P	AR	11<br>4
MD-11	M	32	12	*ABCA4*<br>*ABCA4*	NM_000350	ex22<br>ex22	c.3295T>C<br>c.3259G>A	p.(Ser1099Pro)<br>p.(Glu1087Lys)	HET<br>HET	rs61750119<br>rs61751398	LP<br>P	AR	12<br>4
MD-12	M	25	15	*ABCA4*<br>*ABCA4*<br>*ABCA4*	NM_000350	ex16<br>ex42<br>ex16	c.2461T>A<br>c.5882G>A<br>c.2549A>G	p.(Trp821Arg)<br>p.(Gly1961Glu)<br>p.(Tyr850Cys)	HET<br>HET<br>HET	rs61749433<br>rs1800553<br>rs143797418	P<br>P<br>LP	AR	6<br>4<br>13
MD-13	M	30	6	*ABCA4*<br>*ABCA4*<br>*ABCA4*	NM_000350	ex6<br>ex38<br>ex5	c.634C>T<br>c.5318C>T<br>c.514G>A	p.(Arg212Cys)<br>p.(Ala1773Val)<br>p.(Gly172Ser)	HET<br>HET<br>HET	rs61750200<br>rs760549861<br>rs61748532	P<br>P<br>VUS/LP	AR	14<br>15<br>3
MD-14	M	50	37	*ABCA4*<br>*ABCA4*	NM_000350	int35<br>ex42	c.5018+2T>C<br>c.5882G>A	P.(?)<br>p.(Gly1961Glu)	HET<br>HET	rs61750562<br>rs1800553	P<br>P	AR	16<br>4
**MD-15**	F	30	13	*ABCA4*<br>*ABCA4*	NM_000350	ex33<br>ex42	c.4734_4739delinsCC<br>c.5882G>A	p.(Phe1579Glnfs*8)<br>p.(Gly1961Glu)	HET<br>HET	NA<br>rs1800553	P<br>P	AR	NEW<br>4
**MD-16**	F	22	15	*ABCA4*<br>*ABCA4*	NM_000350	ex12<br>ex21<br>ex40	c.1622T>C<br>c.3113C>T<br>c.5692C>T	p.(Leu541Pro)<br>p.(Ala1038Val)<br>p.(Arg1898Cys)	HET<br>HET<br>HET	rs61751392<br>rs61751374<br>rs201357151	P<br>P<br>VUS/LP	AR	17<br>4<br>18
MD-17	M	56	23	*ABCA4*<br>*ABCA4*	NM_000350	ex28<br>ex42	c.4217del<br>c.5882G>A	p.(His1406Profs*29)<br>p.(Gly1961Glu)	HET<br>HET	NA<br>rs1800553	P<br>P	AR	NEW<br>4
MD-18	F	51	38	*ABCA4*<br>*ABCA4*	NM_000350	ex34<br>ex48	c.4793C>A<br>c.6721C>G	p.(Ala1598Asp)<br>p.(Leu2241Val)	HET<br>HET	rs61750155<br>rs61748521	P<br>LP	AR	1<br>5
MD-19	M	64	NA	*ABCA4*<br>*ABCA4*	NM_000350	ex20<br>ex22	c.2971G>C<br>c.3259G>A	p. (Gly991Arg)<br>p.(Glu1087Lys)	HET<br>HET	rs61750129<br>rs61751398	P<br>P	AR	3<br>4
MD-20	F	17	NA	*ABCA4*<br>*ABCA4*	NM_000350	ex19<br>ex47	c.2888G>T<br>c.6449G>A	p.(Gly963Val)<br>p.(Cys2150Tyr)	HET<br>HET	NA<br>rs61751384	LP<br>P	AR	19<br>20

MD-21	M	53	20	*ABCA4*<br>*ABCA4*<br>*ABCA4*	NM_000350	ex9<br>ex33<br>ex42	c.1222C>T<br>c.4710del<br>c.5882G>A	p.(Arg408*)<br>p.(Ile1571Serfs*10)<br>p.(Gly1961Glu)	HET<br>HET<br>HET	rs61748550<br>rs281865381<br>rs1800553	P<br>LP<br>P	AR	21<br>3<br>4
MD-22	F	52	18	*ABCA4*<br>*ABCA4*<br>*ABCA4*	NM_000350	ex30<br>ex42<br>ex9	c.4462T>C<br>c.5882G>A<br>c.1140T>A	p.(Cys1488Arg)<br>p.(Gly1961Glu)<br>p.(Asn380Lys)	HET<br>HET<br>HET	rs61750146<br>rs1800553<br>rs61748549	P<br>P<br>LP	AR	6<br>4<br>22
MD-23	F	19	9	*ABCA4*	NM_000350	ex3	c.288C>A	p.(Asn96Lys)	HOM	rs886039297	LP	AR	9
**MD-24**	F	20	16	*ABCA4*<br>*ABCA4*	NM_000350	ex38<br>ex42	c.5384T>G<br>c.5882G>A	p.(Leu1795*)<br>p.(Gly1961Glu)	HET<br>HET	NA<br>rs1800553	P<br>P	AR	NEW<br>4
MD-25	M	29	1	*ABCA4*<br>*ABCA4*	NM_000350	ex21<br>ex22	c.3167A>T<br>c.3289A>G	p.(Asn1056Ile)<br>p.(Arg1097Gly)	HET<br>HET	NA<br>NA	LP<br>LP	AR	NEW<br>NEW
MD-26	F	32	NA	*ABCA4*<br>*ABCA4*	NM_000350	ex22<br>ex42	c.3289A>T<br>c.5882G>A	p.(Arg1097*)<br>p.(Gly1961Glu)	HET<br>HET	rs886044731<br>rs1800553	P<br>P	AR	23<br>4
MD-27	M	53	13	*ABCA4*<br>*ABCA4*	NM_000350	ex22<br>ex17	c.3210_3211dupGT<br>c.2640G>C	p.(Ser1071Cysfs*14)<br>p.(Trp880Cys)	HET<br>HET	rs387906385<br>NA	P<br>VUS/LP	AR	4<br>24
MD-28	M	23	15	*ABCA4*<br>*ABCA4*	NM_000350	ex13<br>ex42	c.1819G>A<br>c.5882G>A	p.(Gly607Arg)<br>p.(Gly1961Glu)	HET<br>HET	rs61749412<br>rs1800553	LP<br>P	AR	5<br>4
MD-29	F	14	10	*ABCA4*<br>*ABCA4*	NM_000350	ex1<br>ex19	c.61C>T<br>c.2774G>A	p.(Gln21*)<br>p.(Trp925*)	HET<br>HET	rs770272033<br>NA	P<br>P	AR	11<br>25
MD-30	M	24	13	*ABCA4*<br>*ABCA4*	NM_000350	int30<br>ex36	c.4539+1G>T<br>c.5087G>A	p.(?)<br>p.(Ser1696Asn)	HET<br>HET	rs61751388<br>rs61750564	P<br>P	AR	26<br>6
**MD-31**	M	45	NA	*ABCA4*<br>*ABCA4*<br>*ABCA4*	NM_000350	ex13<br>ex42<br>ex5	*c.1819G>A*<br>c.5882G>A<br>*c.466A>G*	*p.(Gly607Arg)*<br>p.(Gly1961Glu)<br>*p.(Ile156Val)*	HET<br>HET<br>HET	rs61749412<br>rs1800553<br>rs62646863	LP<br>P<br>LP	AR	5<br>4<br>17
MD-32	F	42	35	*ABCA4*<br>*ABCA4*	NM_000350	ex44<br>ex42	c.6088C>T<br>c.5882G>A	p.(Arg2030*)<br>p.(Gly1961Glu)	HET<br>HET	rs61751383<br>rs1800553	P<br>P	AR	6<br>4
**MD-33**	M	21	NA	*ABCA4*<br>*ABCA4*	NM_000350	ex19<br>ex42	c.2768del<br>c.5882G>A	p.(Pro923Glnfs*9)<br>p.(Gly1961Glu)	HET<br>HET	NA<br>rs1800553	LP<br>P	AR	27<br>4
MD-34	M	45	10	*ABCA4*<br>*ABCA4*	NM_000350	ex4<br>ex42	c.428C>T<br>c.5882G>A	p.(Pro143Leu)<br>p.(Gly1961Glu)	HET<br>HET	rs62646860<br>rs1800553	P<br>P	AR	3<br>4
MD-35	F	29	NA	*ABCA4*<br>*ABCA4*	NM_000350	int13<br>int40	c.1937+1G>A<br>c.5714+5G>A	p.(?)<br>p.(Glu1863Leufs*33)	HET<br>HET	rs61752401<br>rs61751407	P<br>P	AR	5<br>28
MD-36	M	33	19	*ABCA4*<br>*ABCA4*<br>*ABCA4*	NM_000350	ex43<br>ex44<br>ex27	c.5908C>T<br>c.6089G>A<br>c.4085G>T	p.(Leu1970Phe)<br>p.(Arg2030Gln)<br>p.(Arg1362Ile)	HET<br>HET<br>HET	rs28938473<br>rs61750641<br>NA	P<br>P<br>VUS/LP	AR	17<br>6<br>NEW
MD-37	M	37	11	*ABCA4*<br>*ABCA4*	NM_000350	ex22<br>ex14	c.3289A>T<br>c.1957C>T	p.(Arg1097*)<br>p.(Arg653Cys)	HET<br>HET	rs886044731<br>rs61749420	P<br>P	AR	23<br>9
MD-38	M	13	NA	*ABCA4*<br>*ABCA4*	NM_000350	int36<br>ex35	c.5196+1G>A<br>c.4919G>A	p.(?)<br>p.(Arg1640Gln)	HET<br>HET	rs61751377<br>rs61751403	P<br>P	AR	4<br>10
MD-39	M	35	14	*ABCA4*<br>*ABCA4*	NM_000350	ex35<br>ex47	c.4956T>G<br>c.6446G>T	p.(Tyr1652*)<br>p.(Arg2149Leu)	HET<br>HET	rs61750561<br>rs61750655	LP<br>LP	AR	12<br>12
**MD-40**	F	37	9	*ABCA4*<br>*ABCA4*	NM_000350	ex13<br>ex42	c.1844T>C<br>c.5882G>A	p.(Val615Ala)<br>p.(Gly1961Glu)	HET<br>HET	NA<br>rs1800553	LP<br>P	AR	29<br>4
MD-41	F	87	12	*ABCA4*<br>*ABCA4*	NM_000350	ex45<br>ex34	c.6184_6188del<br>c.4793C>A	p.(Val2062Argfs*33)<br>p.(Ala1598Asp)	HET<br>HET	NA<br>rs61750155	P<br>P	AR	NEW<br>1
MD-42	F	48	24	*ABCA4*<br>*ABCA4*	NM_000350	ex43<br>ex42	c.5910_5912dup<br>c.5882G>A	p.(Leu1971dup)<br>p.(Gly1961Glu)	HET<br>HET	NA<br>rs1800553	LP<br>P	AR	NEW<br>4

MD-43	F	64	52	*ABCA4*<br>*ABCA4*<br>*ABCA4*	NM_000350	ex19<br>ex42<br>ex16	c.2875A>G<br>c.5882G>A<br>c.2549A>G	p.(Thr959Ala)<br>p.(Gly1961Glu)<br>p.(Tyr850Cys)	HET<br>HET<br>HET	rs368846708<br>rs1800553<br>rs143797418	LP<br>P<br>LP	AR	NEW<br>4<br>13
**MD-44**	F	18	12	*ABCA4*<br>*ABCA4*	NM_000350	ex43<br>ex42	c.5917del<br>c.5882G>A	p.(Val1973*)<br>p.(Gly1961Glu)	HET<br>HET	rs61751389<br>rs1800553	P<br>P	AR	5<br>4
**MD-45**	F	36	24	*ABCA4*<br>*ABCA4*	NM_000350	ex43<br>ex42	c.5959_5964delinsTG<br>c.5882G>A	p.(Gly1987*)<br>p.(Gly1961Glu)	HET<br>HET	NA<br>rs1800553	P<br>P	AR	NEW<br>4
MD-46	M	62	20	*ABCA4*<br>*ABCA4*	NM_000350	ex19<br>ex42	c.2780C>T<br>c.5882G>A	p.(Pro927Leu)<br>p.(Gly1961Glu)	HET<br>HET	NA<br>rs1800553	VUS/LP<br>P	AR	NEW<br>4
MD-47	F	11	6	*ABCA4*<br>*ABCA4*	NM_000350	ex43<br>ex22	c.5917del<br>c.3259G>A	p.(Val1973*)<br>p.(Glu1087Lys)	HET<br>HET	rs61751389<br>rs61751398	P<br>P	AR	5<br>4
MD-48	M	57	12	*ABCA4*<br><br>*ABCA4*	NM_000350	ex27<br><br>ex42	c.3999_4000insACCCCAGA<br>GCCAGAGTGCCAGCCT<br>c.5882G>A	p.(Pro1333_Pro1334insThrPro<br>GluProGluCysGlnPro)<br>p.(Gly1961Glu)	HET<br><br>HET	NA<br><br>rs1800553	LP<br><br>P	AR	NEW<br><br>4
**MD-49**	M	23	18	*ABCA4*<br>*ABCA4*	NM_000350	intron6<br>ex42	c.571-2A>T<br>c.5882G>A	p.(?)<br>p.(Gly1961Glu)	HET<br>HET	rs61748534<br>rs1800553	P<br>P	AR	30<br>4
MD-50	M	58	53	*ABCA4*<br>*ABCA4*	NM_000350	ex14<br>ex48	c.2099G>A<br>c.6721C>G	p.(Trp700*)<br>p.(Leu2241Val)	HET<br>HET	rs61749425<br>rs61748521	P<br>LP	AR	12<br>5
MD-51	M	47	16	*ABCA4*<br>*ABCA4*	NM_000350	ex45<br>ex42	c.6220G>A<br>c.5882G>A	p.(Gly2074Ser)<br>p.(Gly1961Glu)	HET<br>HET	NA<br>rs1800553	LP<br>P	AR	27<br>4
MD-52	M	36	32	*ABCA4*<br>*ABCA4*	NM_000350	int45<br>ex42	c.6282+1G>C<br>c.5882G>A	p.(?)<br>p.(Gly1961Glu)	HET<br>HET	rs770453727<br>rs1800553	P<br>P	AR	27<br>4
MD-53	M	60	53	*ABCA4*<br>*ABCA4*	NM_000350	ex12<br>ex42	c.1757A>G<br>c.5882G>A	p.(Asp586Gly)<br>p.(Gly1961Glu)	HET<br>HET	rs1553192682<br>rs1800553	LP<br>P	AR	31<br>4
MD-54	F	34	19	*ABCA4*<br>*ABCA4*	NM_000350	ex43<br>ex44	c.5942C>G<br>c.6089G>A	p.(Thr1981Arg)<br>p.(Arg2030Gln)	HET<br>HET	rs752147871<br>rs61750641	P<br>P	AR	7<br>6
MD-55	F	14	11	*ABCA4*<br>*ABCA4*	NM_000350	intron6<br>ex42	c.571-2A>T<br>c.5882G>A	p.(?)<br>p.(Gly1961Glu)	HET<br>HET	rs61748534<br>rs1800553	P<br>P	AR	30<br>4
MD-56	F	16	12	*ABCA4*<br>*ABCA4*	NM_000350	ex44<br>int40	c.6088C>T<br>c.5714+5G>A	p.(Arg2030*)<br>p.(Glu1863Leufs*33)	HET<br>HET	rs61751383<br>rs61751407	P<br>P	AR	6<br>28
MD-57	F	46	35	*ABCA4*<br>*ABCA4*	NM_000350	ex19<br>ex42	c.2860T>G<br>c.5882G>A	p.(Tyr954Asp)<br>p.(Gly1961Glu)	HET<br>HET	rs61749447<br>rs1800553	LP<br>P	AR	12<br>4
**MD-58**	F	39	17	*ABCA4*<br>*ABCA4*	NM_000350	ex35<br>ex34	c.4956T>G<br>c.4793C>A	p.(Tyr1652*)<br>p.(Ala1598Asp)	HET<br>HET	rs61750561<br>rs61750155	LP<br>P	AR	12<br>1
MD-59	M	37	6	*ABCA4*<br>*ABCA4*	NM_000350	ex21<br>ex42	c.3064G>A<br>c.5882G>A	p.(Glu1022Lys)<br>p.(Gly1961Glu)	HET<br>HET	rs61749459<br>rs1800553	P<br>P	AR	12<br>4
MD-60	F	75	55	*ABCA4*<br>*ABCA4*	NM_000350	ex3<br>ex42	c.247_250dup<br>c.5882G>A	p.(Ser84Thrfs*16)<br>p.(Gly1961Glu)	HET<br>HET	rs1005271380<br>rs1800553	P<br>P	AR	10<br>4
**MD-61**	M	14	7	*ABCA4*<br>*ABCA4*	NM_000350	ex3<br>ex28	c.247_250dup<br>c.4139C>T	p.(Ser84Thrfs*16)<br>p.(Pro1380Leu)	HET<br>HET	rs1005271380<br>rs61750130	P<br>P	AR	10<br>6
**MD-62**	F	8	5	*BEST1*	NM_004183	ex7	c.718_720dup	p.(Val240dup)	HET	NA	LP	AD	NEW
MD-63	M	65	62	*BEST1*	NM_004183	ex4	c.436G>A	p.(Ala146Thr)	HET	rs1237501081	LP	AD	32
MD-64	M	44	NA	*BEST1*	NM_004183	ex4	c.278G>C	p.(Trp93Ser)	HET	NA	P	AD	33
MD-65	M	44	9	*BEST1*	NM_004183	ex6	c.652C>A	p.(Arg218Ser)	HET	rs281865238	P	AD	34

MD-66	F	15	8	*BEST1*	NM_004183	ex5	c.544T>C	p.(Trp182Arg)	HET	rs1565390925	LP	AD	33
MD-67	F	15	4	*BEST1*	NM_004183	ex2	c.44G>A	p.(Gly15Asp)	HET	rs766379510	LP	AD	35
MD-68	F	15	NA	*BEST1*	NM_004183	ex6	c.652C>T	p.(Arg218Cys)	HET	rs281865238	P	AD	34
MD-69	M	55	46	*CFI*	NM_000204	ex13	c.1573T>C	p.(Ser525Pro)	HET	NA	VUS/LP	AD	NEW
MD-70	F	47	8	*GUCY2D*	NM_000180	ex13	c.2480A>C	p.(Tyr827Ser)	HET	NA	VUS/LP	AD	NEW
MD-71	F	54	45	*PRPH2*	NM_000322	ex2	c.583C>T	p.(Arg195*)	HET	rs1322278463	P	AD	36
MD-72	F	49	38	*PRPH2*	NM_000322	ex2	c.652T>C	p.(Ser218Pro)	HET	rs1582764878	LP	AD	37
MD-73	F	50	44	*PRPH2*	NM_000322	ex2	c.668T>A	p.(Ile223Asn)	HET	NA	P	AD	38
MD-74	M	57	39	*PRPH2*	NM_000322	ex1	c.515G>A	p.(Arg172Gln)	HET	rs61755793	P	AD	39
MD-75	M	56	NA	*PRPH2*	NM_000322	ex1	c.499G>A	p.(Gly167Ser)	HET	rs527236098	P	AD	40
**MD-76**	M	54	17	*PRPH2*	NM_000322	ex1	c.514C>T	p.(Arg172Trp)	HET	rs61755792	P	AD	39
**MD-77**	F	53	48	*PRPH2*	NM_000322	ex1	c.499G>A	p.(Gly167Ser)	HET	rs527236098	P	AD	40
MD-78	F	50	41	*PRPH2*	NM_000322	ex1	c.515G>A	p.(Arg172Gln)	HET	rs61755793	P	AD	39
MD-79	M	50	46	*PRPH2*	NM_000322	ex1	c.499G>A	p.(Gly167Ser)	HET	rs527236098	P	AD	40
MD-80	F	45	34	*RP1L1*	NM_178857	ex2	c.563T>C	p.(Leu188Pro)	HET	NA	VUS/LP	AD	NEW

Legend: AD, autosomal dominant; AR, autosomal recessive; HET, heterozygous; HOM, homozygous; F, female; M, male; ex, exon; int, intron; in **bold**, segregation performed; in *italics*, *in cis* variants; NA, not available; UNK, unknown; VUS, variant of unknown significance; LP, likely pathogenic; P, pathogenic.

Table S3. Demographic and genetic characteristics of CD and CRD probands.

ID	Sex	Age	Genetic class	ONSET	Gene	RefSeq	Exon/ intron	Nucleotide change	Amino acid change	Allele state	dbSNP rs	VARSOME	Transmission	REF
CD/CRD-1	F	66	CD	8	*ABCA4*	NM_000350	ex34	c.4793C>A	p.(Ala1598Asp)	HET	rs61750155	P	AR	1
					ABCA4		ex42	c.5882G>A	p.(Gly1961Glu)	HET	rs1800553	P		4
					ABCA4		ex3	c.288C>A	p.(Asn96Lys)	HET	rs886039297	LP		9
CD/CRD-2	F	44	CRD	NA	*ABCA4*	NM_000350	ex10	c.52C>T	p.(Arg18Trp)	HET	rs121909205	P	AR	14
					ABCA4		ex15	c.2300T>A	p.(Val767Asp)	HET	rs61751395	P		10
CD/CRD-3	F	67	CRD	NA	*ABCA4*	NM_000350	ex12	c.1622T>C	p.(Leu541Pro)	HET	rs61751392	P	AR	17
					ABCA4		ex21	c.3113C>T	p.(Ala1038Val)	HET	rs61751374	P		4
					ABCA4		ex11	c.1522C>T	p.(Arg508Cys)	HET	rs138157885	VUS/LP		41
					ABCA4		ex37	c.5261A>G	p.(Tyr1754Cys)	HET	NA	VUS/LP		27
CD/CRD-4	M	65	CRD	15	*ABCA4*	NM_000350	ex34	c.4793C>A	p.(Ala1598Asp)	HET	rs61750155	P	AR	1
					ABCA4		ex31	c.4577C>T	p.(Thr1526Met)	HET	rs61750152	P		17
CD/CRD-5	F	25	CD	17	*ABCA4*	NM_000350	ex43	c.5961_5964del	p.(Asp1988Profs*3)	HET	rs61750640	P	AR	12
					ABCA4		ex42	c.5882G>A	p.(Gly1961Glu)	HET	rs1800553	P		4
					ABCA4		ex43	c.5959G>T	p.(Gly1987Trp)	HET	NA	LP		NEW
CD/CRD-6	F	38	CD	11	*ABCA4*	NM_000350	ex29	c.4346G>A	p.(Trp1449*)	HET	rs61750143	P	AR	6
					ABCA4		ex25	c.3806T>C	p.(Leu1269Pro)	HET	NA	VUS/LP		27
CD/CRD-7	M	67	CRD	16	*ABCA4*	NM_000350	ex14	c.1957C>T	p.(Arg653Cys)	HET	rs61749420	P	AR	9
					ABCA4		ex37	c.5285C>A	p.(Ala1762Asp)	HET	rs121909206	LP		9
**CD/CRD-8**	F	8	CD	5	*ABCA4*	NM_000350	int35	c.5018+2T>C	p.(?)	HET	rs61750562	P	AR	16
					ABCA4		ex47	c.6449G>A	p.(Cys2150Tyr)	HET	rs61751384	P		20
CD/CRD-9	M	66	CD	50	*ABCA4*	NM_000350	ex1-5	c.1-?_571-?del	p.(?)	HET	NA	P	AR	NEW
					ABCA4		ex38	c.5329A>T	p.(Met1777Leu)	HET	rs375184282	VUS/LP		27
**CD/CRD-10**	M	16	CD	5	*ABCA4*	NM_000350	ex12	*c.1622T>C*	*p.(Leu541Pro)*	HET	rs61751392	P	AR	17
					ABCA4		int9	c.1239+1G>C	p.(?)	HET	rs765707028	P		
					ABCA4		ex21	*c.3113C>T*	*p.(Ala1038Val)*	HET	rs61751374	P		4
CD/CRD-11	F	42	CD	21	*ABCA4*	NM_000350	int5	c.571-1G>T	p.(?)	HET	rs61748533	P	AR	12
					ABCA4		ex36	c.5087G>A	p.(Ser1696Asn)	HET	rs61750564	P		6
CD/CRD-12	F	16	CRD	13	*ABCA4*	NM_000350	int36	c.5196+1G>A	p.(?)	HET	rs61751377	P	AR	4
					ABCA4		*ex25*	c.3813G>C	p.(Glu1271Asp)	HET	NA	P		
**CD/CRD-13**	F	53	CRD	20	*ABCA4*	NM_000350	ex4	c.428C>T	p.(Pro143Leu)	HET	rs62646860	P	AR	3
					ABCA4		ex42	c.5882G>A	p.(Gly1961Glu)	HET	rs1800553	P		4
CD/CRD-14	F	52	CD	5	*ABCA4*	NM_000350	ex4	c.428C>T	p.(Pro143Leu)	HOM	rs62646860	P	AR	3
CD/CRD-15	M	28	CRD	6	*ABCA4*	NM_000350	ex43	c.5917del	p.(Val1973*)	HET	rs61751389	P	AR	5
					ABCA4		ex22	c.3259G>A	p.(Glu1087Lys)	HET	rs61751398	P		4
CD/CRD-16	M	40	CD	12	*ABCA4*	NM_000350	int35	c.5018+2T>C	p.(?)	HET	rs61750562	P	AR	16
					ABCA4		ex42	c.5882G>A	p.(Gly1961Glu)	HET	rs1800553	P		4
					ABCA4		ex16	c.2549A>G	p.(Tyr850Cys)	HET	rs143797418	LP		13
CD/CRD-17	M	24	CD	11	*ABCA4*	NM_000350	ex8	c.1085_1086del	p.(Tyr362*)	HET	NA	P	AR	NEW
					ABCA4		ex17	c.2624T>C	p.(Leu875Pro)	HET	NA	VUS/LP		NEW
CD/CRD-18	F	63	CRD	9	*ABCA4*	NM_000350	ex47	c.6445C>T	p.(Arg2149*)	HET	rs61750654	P	AR	6
					ABCA4		ex23	c.(3192-90_3418)_(3418_3562)dup	p.(?)	HET	NA	P		NEW
**CD/CRD-19**	F	52	CRD	30	*ABCA4*	NM_000350	int13	c.1937+1G>A	p.(?)	HET	rs61752401	P	AR	5
					ABCA4		ex42	c.5882G>A	p.(Gly1961Glu)	HET	rs1800553	P		4
**CD/CRD-20**	M	59	CD	12	*ABCA4*	NM_000350	ex43	c.5959_5964delinsTG	p.(Gly1987*)	HET	NA	P	AR	NEW
					ABCA4		int40	c.5714+5G>A	p.(Glu1863Leufs*33)	HET	rs61751407	P		28
CD/CRD-21	M	9	CRD	1	*ADAM9*	NM_003816	ex8	c.725T>G	p.(Leu242Arg)	HOM	NA	VUS/LP	AR	NEW
CD/CRD-22	M	29	CRD	1	*BEST1*	NM_004183	ex4	c.318dup	p.(Met107Hisfs*125)	HET	NA	P	AD	NEW
CD/CRD-23	M	47	CRD	19	*CACNA1F*	NM_005183	int23	c.2874-1G>C	p.(?)	HEM	NA	P	XL	NEW
CD/CRD-24	M	22	CRD	3	*CACNA1F*	NM_005183	ex23	c.2804_2806del	p.(Phe935del)	HEM	rs782068089	VUS/LP	XL	NEW
CD/CRD-25	F	48	CRD	44	*CDHR1*	NM_033100	ex8	c.783G>A	p.(Pro261=)	HET	rs147346345	VUS/LP	AR	42
					CDHR1		ex13	c.1373T>A	p.(Val458Asp)	HET	rs760942217	VUS/LP		43
CD/CRD-26	M	52	CRD	28	*CDHR1*	NM_033100	ex17	c.2522_2528del	p.(Ile841Serfs*119)	HET	rs794727197	P	AR	44
					CDHR1		ex8	c.783G>A	p.(Pro261=)	HET	rs147346345	VUS/LP		42

CD/CRD-27	M	70	CRD	62	*CFH*	NM_000186	ex16	c.2440C>T	p.(Pro814Ser)	HET	NA	VUS/LP	AD	NEW
CD/CRD-28	F	39	CD	1	*CNGA3*	NM_001298	ex7	c.572G>A	p.(Cys191Tyr)	HET	rs761554853	P	AR	45
					CNGA3		ex8	c.848G>A	p.(Arg283Gln)	HET	rs104893614	P		45
**CD/CRD-29**	F	15	CD	NA	*CNGA3*	NM_001298	ex8	c.1279C>T	p.(Arg427Cys)	HET	rs141386891	P	AR	46
					CNGA3		ex8	c.940_942del	p.(Ile314del)	HET	rs777878533	P		46
**CD/CRD-30**	F	39	CD	7	*CNGB3*	NM_019098	ex10	c.1148del	p.(Thr383Ilefs*13)	HOM	rs397515360	P	AR	47
CD/CRD-31	F	44	CD	6	*CNGB3*	NM_019098	ex2	c.143del	p.(Gly48Valfs*35)	HOM	NA	LP	AR	NEW
CD/CRD-32	F	29	CRD	6	*CRB1*	NM_201253	ex7	c.2149G>T	p.(Gly717Cys)	HET	NA	LP	AD	NEW
CD/CRD-33	F	60	CD	52	*CRX*	NM_000554	ex3	c.166G>A	p.(Ala56Thr)	HET	rs61748437	LP	AD	48
CD/CRD-34	M	68	CRD	56	*CRX*	NM_000554	ex4	c.329del	p.(Gly110Alafs*77)	HET	rs761108522	P	AD	NEW
CD/CRD-35	F	56	CRD	20	GUCA1A	NM_000409	ex4	c.312_313delinsGC	p.(Asn104_Gly105delinsLysArg)	HET	NA	VUS/LP	AD	NEW
CD/CRD-36	M	51	CRD	18	*GUCY2D*	NM_000180	ex13	c.2512C>T	p.(Arg838Cys)	HET	rs61750172	P	AD	49
CD/CRD-37	M	43	CD	25	*GUCY2D*	NM_000180	ex13	c.2512 C>T	p.(Arg838Cys)	HOM	rs61750172	P	AR	49
**CD/CRD-38**	F	12	CD	5	*GUCY2D*	NM_000180	ex13	c.2512C>T	p.(Arg838Cys)	HET	rs61750172	P	AD	49
**CD/CRD-39**	M	77	CD	65	*GUCY2D*	NM_000180	ex2	c.286T>C	p.(Phe96Leu)	HET	NA	VUS/LP	AD	NEW
CD/CRD-40	M	26	CD	3	*GUCY2D*	NM_000180	ex13	c.2546C>G	p.(Thr849Arg)	HET	NA	LP	AD	NEW
**CD/CRD-41**	F	47	CRD	35	*IMPG2*	NM_016247	ex2	c.283G>C	p.(Glu95Gln)	HET	rs1198094357	VUS/LP	AD	NEW
CD/CRD-42	F	39	CD	14	*KCNV2*	NM_133497	ex2	c.1381G>T	p.(Gly461*)	HOM	rs149648640	P	AR	50
CD/CRD-43	F	30	CD	3	*KCNV2*	NM_133497	ex2	c.1427T>G	p.(Leu476Arg)	HOM	rs796658305	LP	AR	NEW
CD/CRD-44	M	40	CD	31	*PDE6C*	NM_006204	int20	c.2367+1_2367+5del	p.(?)	HET	rs796051871	P	AR	NEW
					PDE6C		ex17	c.2087C>T	p.(Thr696Met)	HET	rs41290222	VUS/LP		NEW
CD/CRD-45	M	57	CRD	25	*POC1B*	NM_172240	ex4	c.317G>C	p.(Arg106Pro)	HET	rs76216585	LP	AR	51
					POC1B		ex6	c.587C>T	p.(Pro196Leu)	HET	NA	VUS/LP		NEW
CD/CRD-46	M	68	CD	30	*PRPH2*	NM_000322	ex2	c.621C>A	p.(Asp207Glu)	HET	NA	LP	AD	NEW
CD/CRD-47	F	52	CD	40	*PRPH2*	NM_000322	ex1	c.514C>T	p.(Arg172Trp)	HET	rs61755792	P	AD	39
**CD/CRD-48**	F	63	CD	10	*PRPH2*	NM_000322	ex1	c.568A>G	p.(Lys190Glu)	HET	NA	VUS/LP	AD	NEW
CD/CRD-49	F	64	CD	7	*RAB28*	NM_001017979	ex4	c.321G>A	p.(Trp107*)	HET	NA	P	AD	52
**CD/CRD-50**	M	62	CRD	16	*RPGR*	NM_001034853	ex15	c.3178_3179del	p.(Glu1060Argfs*18)	HEM	rs771214648	P	XL	53
**CD/CRD-51**	M	46	CD	25	*RPGR*	NM_001034853	ex15	c.3317dup	p.(Ser1107Valfs*4)	HEM	rs886041376	P	XL	54
**CD/CRD-52**	M	37	CRD	24	*RPGR*	NM_001034853	ex15	c.2236_2237del	p.(Glu746Argfs*23)	HEM	rs1555961852	P	XL	55
**CD/CRD-53**	M	24	CRD	12	*TTLL5*	NM_015072	ex13	c.1060G>A	p.(Val354Met)	HOM	rs781509883	VUS/LP	AR	NEW
CD/CRD-54	M	71	CRD	60	*TTLL5*	NM_015072	ex17	c.1442G>C	p.(Gly481Ala)	HOM	rs771482604	VUS/LP	AR	NEW
CD/CRD-55	M	41	CRD	34	*TTLL5*	NM_015072	ex10	c.800T>C	p.(Leu267Pro)	HOM	NA	VUS/LP	AR	NEW
CD/CRD-56	F	7	CRD	1	*TULP1*	NM_003322	ex10	c.901C>T	p.(Gln301*)	HET	rs201070350	P	AR	56
					TULP1		ex8	c.822G>T	p.(Lys274Asn)	HET	NA	P		NEW

Legend: AD, autosomal dominant; AR, autosomal recessive; XL, X-linked; HET, heterozygous; HOM, homozygous; HEM, hemizygous; F, female; M, male; ex, exon; int, intron; in **bold**, segregation performed; in *italics*, *in cis* variants; NA, not available; UNK, unknown; VUS, variant of unknown significance; LP, likely pathogenic; P, pathogenic.

1. Maugeri, A. *et al*. Mutations in the ABCA4 (ABCR) gene are the major cause of autosomal recessive cone-rod dystrophy. *Am J Hum Genet*. **67**, 960-966 (2000).
2. Allikmets, R., Zernant, J., Lee, W. Penetrance of the ABCA4 p.Asn1868Ile Allele in Stargardt Disease. *Invest Ophthalmol Vis Sci*. **59**, 5564-5565 (2018).
3. Jaakson, K. *et al*. Genotyping microarray (gene chip) for the ABCR (ABCA4) gene. *Hum Mutat*. **22**, 395-403 (2003).
4. Allikmets, R. *et al*. A photoreceptor cell-specific ATP-binding transporter gene (ABCR) is mutated in recessive Stargardt macular dystrophy. *Nat Genet*. **15**, 236-246 (1997).
5. Rivera, A. *et al*. A comprehensive survey of sequence variation in the ABCA4 (ABCR) gene in Stargardt disease and age-related macular degeneration. *Am J Hum Genet*. **67**, 800-813 (2000).
6. Lewis, R.A. *et al*. Genotype/Phenotype analysis of a photoreceptor-specific ATP-binding cassette transporter gene, ABCR, in Stargardt disease. *Am J Hum Genet*. **64**, 422-434 (1999).
7. Schulz, H.L. *et al*. Mutation Spectrum of the ABCA4 Gene in 335 Stargardt Disease Patients From a Multicenter German Cohort-Impact of Selected Deep Intronic Variants and Common SNPs. *Invest Ophthalmol Vis Sci*. **58**, 394-403 (2017).
8. Nassisi, M. *et al*. Expanding the Mutation Spectrum in ABCA4: Sixty Novel Disease Causing Variants and Their Associated Phenotype in a Large French Stargardt Cohort. *Int J Mol Sci*. **19**, 2196 (2018).
9. Stenirri, S. *et al*. Denaturing HPLC profiling of the ABCA4 gene for reliable detection of allelic variations. *Clin Chem*. **50**, 1336-1343 (2004).

10. Simonelli, F. *et al*. New ABCR mutations and clinical phenotype in Italian patients with Stargardt disease. *Invest Ophthalmol Vis Sci*. **41**, 892-897 (2000).

11. Sodi, A. *et al*. Different patterns of fundus autofluorescence related to ABCA4 gene mutations in Stargardt disease. *Ophthalmic Surg Lasers Imaging*. **41**, 48-53 (2010).

12. Fumagalli, A. *et al*. Mutational scanning of the ABCR gene with double-gradient denaturing-gradient gel electrophoresis (DG-DGGE) in Italian Stargardt disease patients. *Hum Genet*. **109**, 326-338 (2001).

13. Oldani, M. *et al*. Clinical and molecular genetic study of 12 Italian families with autosomal recessive Stargardt disease. *Genet Mol Res*. **11**, 4342-4350 (2012).

14. Gerber, S. *et al*. Complete exon-intron structure of the retina-specific ATP binding transporter gene (ABCR) allows the identification of novel mutations underlying Stargardt disease. *Genomics*. **48**, 139-142 (1998).

15. Stenirri, S. *et al*. Are microarrays useful in the screening of ABCA4 mutations in Italian patients affected by macular degenerations? *Clin Chem Lab Med*. **46**, 1250-1255 (2008).

16. Cideciyan, A.V. *et al*. ABCA4 disease progression and a proposed strategy for gene therapy. *Hum Mol Genet*. **18**, 931-941 (2009).

17. Rozet, J.M. *et al*. Spectrum of ABCR gene mutations in autosomal recessive macular dystrophies. *Eur J Hum Genet*. **6**, 291-295 (1998).

18. Zaneveld, J. *et al*. Comprehensive analysis of patients with Stargardt macular dystrophy reveals new genotype-phenotype correlations and unexpected diagnostic revisions. *Genet Med*. **17**, 262-270 (2015).

19. Sodi, A. *et al*. En face OCT in Stargardt disease. *Graefes Arch Clin Exp Ophthalmol*. **254**, 1669-1679 (2016).

20. Fishman, G.A. *et al*. Variation of clinical expression in patients with Stargardt dystrophy and sequence variations in the ABCR gene. *Arch Ophthalmol*. **117**, 504-510 (1999).

21. Birch, D.G. *et al*. Visual function in patients with cone-rod dystrophy (CRD) associated with mutations in the ABCA4(ABCR) gene. *Exp Eye Res*. **73**, 877-886 (2001).

22. Riveiro-Alvarez, R. *et al*. Frequency of ABCA4 mutations in 278 Spanish controls: an insight into the prevalence of autosomal recessive Stargardt disease. *Br J Ophthalmol*. **93**, 1359-1364 (2009).

23. Fujinami, K. *et al*. ABCA4 gene screening by next-generation sequencing in a British cohort. *Invest Ophthalmol Vis Sci*. **54**, 6662-6674 (2013).

24. Abed, E. *et al*. Correlation of Macular Focal Electroretinogram with Ellipsoid Zone Extension in Stargardt Disease. *J Ophthalmol*. **2017**, 3643495 (2017).

25. Hu, F.Y. *et al*. ABCA4 Gene Screening in a Chinese Cohort With Stargardt Disease: Identification of 37 Novel Variants. *Front Genet*. **10**, 773 (2019).

26. Cremers, F.P. *et al*. Autosomal recessive retinitis pigmentosa and cone-rod dystrophy caused by splice site mutations in the Stargardt's disease gene ABCR. *Hum Mol Genet*. **7**, 355-362 (1998).

27. Abed, E. *et al*. Early impairment of the full-field photopic negative response in patients with Stargardt disease and pathogenic variants of the ABCA4 gene. *Clin Exp Ophthalmol*. **46**, 519-530 (2018).

28. Sangermano, R. *et al*. ABCA4 midigenes reveal the full splice spectrum of all reported noncanonical splice site variants in Stargardt disease. *Genome Res*. **28**, 100-110 (2018).
29. Paavo, M. *et al*. Mutations in GPR143/OA1 and ABCA4 Inform Interpretations of Short-Wavelength and Near-Infrared Fundus Autofluorescence. *Invest Ophthalmol Vis Sci*. **59**, 2459-2469 (2018).
30. Rossi, S. *et al*. Subretinal Fibrosis in Stargardt's Disease with Fundus Flavimaculatus and ABCA4 Gene Mutation. *Case Rep Ophthalmol*. **3**, 410-417 (2012).
31. Fujinami, K. *et al*. Clinical and molecular characteristics of childhood-onset Stargardt disease. *Ophthalmology*. **122**, 326-334 (2015).
32. Gao, T. *et al*. Clinical and Mutation Analysis of Patients with Best Vitelliform Macular Dystrophy or Autosomal Recessive Bestrophinopathy in Chinese Population. *Biomed Res Int*. **2018**, 4582816 (2018).
33. Frecer, V. *et al*. Pathogenicity of new BEST1 variants identified in Italian patients with best vitelliform macular dystrophy assessed by computational structural biology. *J Transl Med*. **17**, 330 (2019).
34. Bakall, B. *et al*. The mutation spectrum of the bestrophin protein--functional implications. *Hum Genet*. **104**, 383-389 (1999).
35. Querques, G. *et al*. The spectrum of subclinical Best vitelliform macular dystrophy in subjects with mutations in BEST1 gene. *Invest Ophthalmol Vis Sci*. **52**, 4678-4684 (2011).
36. Alapati, A. *et al*. Molecular diagnostic testing by eyeGENE: analysis of patients with hereditary retinal dystrophy phenotypes involving central vision loss. *Invest Ophthalmol Vis Sci*. **55**, 5510-5521 (2014).

37. Jespersgaard, C. *et al*. Molecular genetic analysis using targeted NGS analysis of 677 individuals with retinal dystrophy. *Sci Rep*. **9**, 1219 (2019).

38. Strafella, C. *et al*. NGS Analysis for Molecular Diagnosis of Retinitis Pigmentosa (RP): Detection of a Novel Variant in PRPH2 Gene. *Genes (Basel)*. **10**, 792 (2019).

39. Wells, J. *et al*. Mutations in the human retinal degeneration slow (RDS) gene can cause either retinitis pigmentosa or macular dystrophy. *Nat Genet*. **3**, 213-218 (1993).

40. Testa, F. *et al*. A novel mutation in the RDS gene in an Italian family with pattern dystrophy. *Br J Ophthalmol*. **89**, 1066-1068 (2005).

41. Michaelides, M. *et al*. ABCA4 mutations and discordant ABCA4 alleles in patients and siblings with bull's-eye maculopathy. *Br J Ophthalmol*. **91**, 1650-1655 (2007).

42. Glöckle, N. *et al*. Panel-based next generation sequencing as a reliable and efficient technique to detect mutations in unselected patients with retinal dystrophies. *Eur J Hum Genet*. **22**, 99-104 (2014).

43. Ba-Abbad, R. *et al*. A clinical study of patients with novel CDHR1 genotypes associated with late-onset macular dystrophy. *Eye (Lond)*. **35**, 1482-1489 (2021).

44. Weisschuh, N. *et al*. Mutation Detection in Patients with Retinal Dystrophies Using Targeted Next Generation Sequencing. *PLoS One*. **11**, e0145951 (2016).

45. Muraki-Oda, S. *et al*. Functional analysis of rod monochromacy-associated missense mutations in the CNGA3 subunit of the cone photoreceptor cGMP-gated channel. *Biochem Biophys Res Commun*. **362**, 88-93 (2007).

46. Wissinger, B. *et al*. CNGA3 mutations in hereditary cone photoreceptor disorders. *Am J Hum Genet*. **69**, 722-737 (2001).

47. Sundin, O.H. *et al*. Genetic basis of total colourblindness among the Pingelapese islanders. *Nat Genet*. **25**, 289-293 (2000).

48. Lotery, A.J. *et al*. Mutation analysis of 3 genes in patients with Leber congenital amaurosis. *Arch Ophthalmol*. **118**, 538-543 (2000).

49. Kelsell, R.E. *et al*. Mutations in the retinal guanylate cyclase (RETGC-1) gene in dominant cone-rod dystrophy. *Hum Mol Genet*. **7**, 1179-1184 (1998).

50. Robson, A.G. *et al*. "Cone dystrophy with supernormal rod electroretinogram": a comprehensive genotype/phenotype study including fundus autofluorescence and extensive electrophysiology. *Retina*. **30**, 51-62 (2010).

51. Roosing, S. *et al*. Disruption of the basal body protein POC1B results in autosomal-recessive cone-rod dystrophy. *Am J Hum Genet*. **95**, 131-142 (2014).

52. Iarossi, G. *et al*. Expanding the Clinical and Genetic Spectrum of RAB28-Related Cone-Rod Dystrophy: Pathogenicity of Novel Variants in Italian Families. *Int J Mol Sci*. **22**, 381 (2020).

53. Wang, J. *et al*. Dependable and efficient clinical utility of target capture-based deep sequencing in molecular diagnosis of retinitis pigmentosa. *Invest Ophthalmol Vis Sci*. **55**, 6213-6223 (2014).

54. Thiadens, A.A. *et al*. Clinical course of cone dystrophy caused by mutations in the RPGR gene. *Graefes Arch Clin Exp Ophthalmol*. **249**, 1527-1535 (2011).

55. Vervoort, R. *et al*. Mutational hot spot within a new RPGR exon in X-linked retinitis pigmentosa. *Nat Genet*. **25**, 462-466 (2000).

56. Li, Y. *et al*. Mutation survey of known LCA genes and loci in the Saudi Arabian population. *Invest Ophthalmol Vis Sci*. **50**, 1336-1343 (2009).
